# SMARCA4 activation engages FOSL1 to drive enhancer reprogramming and tumorigenic phenotypes in SMARCA4-deficient LUAD cells

**DOI:** 10.1038/s41420-026-03100-3

**Published:** 2026-04-20

**Authors:** Hye-Ju Yang, Eun-Ju Kim, Sungho Kim, Sang-Hyun Song, Tae-You Kim

**Affiliations:** 1 Department of Molecular Medicine and Biopharmaceutical Sciences, Graduate School of Convergence Science and Technology, Seoul National University, Seoul, Republic of Korea; 2 Cancer Genomics Research Laboratory, Cancer Research Institute, Seoul National University, Seoul, Republic of Korea; 3 State University of New York Upstate Medical University, Syracuse, NY USA; 4 Department of Internal Medicine, Seoul National University Hospital, Seoul, Republic of Korea

**Keywords:** Cancer epigenetics, Chromatin remodelling, Epigenetics

## Abstract

SMARCA4, the ATPase component of the SWI/SNF chromatin remodeling complex, is integral to the regulation of gene expression through modulation of chromatin accessibility. Although SMARCA4 is frequently inactivated in lung adenocarcinoma (LUAD), a subset of tumors exhibits elevated SMARCA4 expression, suggesting a context-dependent oncogenic function. However, the molecular mechanisms by which elevated SMARCA4 exerts oncogenic functions in LUAD remain unclear. Here, using a SMARCA4-deficient LUAD cellular model, we show that SMARCA4 overexpression reorganizes enhancer landscapes and establishes a cooperative transcriptional network involving FOSL1, thereby promoting cancer cell proliferation and tumorigenic phenotypes. Integrative multi-omics analyses revealed that SMARCA4 directly cooperates with FOSL1 at active enhancers, leading to the activation of tumor-associated transcriptional programs. Functionally, genetic depletion of FOSL1 or pharmacological inhibition of SMARCA4 reduced cell proliferation and migration and suppressed tumor growth in vitro and in vivo. Importantly, high co-expression of SMARCA4 and FOSL1 was associated with poor clinical outcomes in LUAD patient cohorts. Together, these findings define an epigenetic regulatory axis between SMARCA4 and FOSL1 induced by SMARCA4 activation in SMARCA4-deficient LUAD cells, thereby providing mechanistic insight into how SMARCA4 activates oncogenic regulatory programs in this specific cellular context.

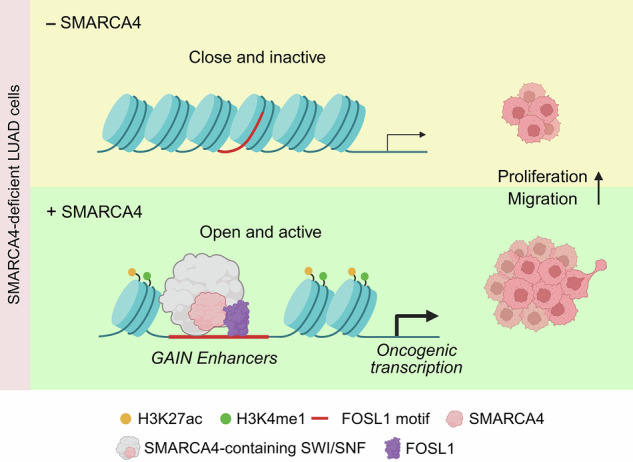

## Introduction

The mammalian SWI/SNF (BAF) complexes exhibit ATP-dependent chromatin remodeling capabilities and are integral to DNA repair, transcriptional regulation, and developmental processes [1, 2]. These complexes predominantly interact with distal regulatory elements, such as enhancers, in order to preserve an open chromatin state and modulate gene expression [1, 3–5]. Composed of multiple core subunits, the SWI/SNF complexes include SMARCB1, SMARCC1, and the mutually exclusive ATPase subunits SMARCA4 and SMARCA2 [6–8]. Comprehensive genome-wide sequencing studies have revealed that genes encoding the subunits of the SWI/SNF complex are frequently subjected to mutations in human malignancies, with such genetic alterations collectively present in approximately 25% of cases [2, 6, 9].

SMARCA4, a pivotal ATPase subunit of the SWI/SNF complex, enhances chromatin accessibility by repositioning nucleosomes through the utilization of energy derived from ATP hydrolysis [1, 10, 11]. Recent investigations have suggested that SMARCA4 may function as either a tumor suppressor or an oncogenic factor, depending on the specific biological context [12–15]. Notably, SMARCA4 is markedly overexpressed in a variety of cancers, including lung adenocarcinoma (LUAD), breast carcinoma, colorectal cancer, and prostate cancer. The upregulation of SMARCA4 correlates with a diminished prognosis, implicating its role as an oncogene [16–18]. Nevertheless, the oncogenic mechanisms by which SMARCA4 influences LUAD remain inadequately elucidated, necessitating further research to clarify how it modulates the epigenetic and transcriptional framework to facilitate tumorigenesis.

Enhancers represent distal DNA elements that govern gene expression in a temporally and spatially precise manner, thereby serving critical functions in developmental and biological processes [19, 20]. Active enhancers are characterized by an open chromatin architecture and a pronounced enrichment of active histone modifications, such as histone H3 lysine 27 acetylation (H3K27ac) and histone H3 lysine 4 mono-methylation (H3K4me1) [21–23], which can be identified through methodologies such as chromatin immunoprecipitation sequencing (ChIP-seq) or chromatin accessibility assays like the assay for transposase-accessible chromatin (ATAC-seq) [24].

Enhancers harbor binding sites for transcription factors (TFs), which are instrumental in the activation of target genes [19, 25, 26]. TFs engage with enhancers and subsequently recruit chromatin-remodeling complexes (e.g., SWI/SNF, CHD) as well as histone-modifying enzymes (e.g., p300/CBP, MLL3/4), thereby facilitating chromatin accessibility and establishing a distinctive array of histone modifications on adjacent nucleosomes [25, 27, 28]. Although enhancers make up only a small portion of the genome, SWI/SNF complexes are highly enriched at these regions and play a crucial role in regulating enhancer accessibility, which is necessary for TFs to activate gene expression [9, 21, 29].

Modifications or reprogramming of enhancers have emerged as fundamental characteristics of cancer, as the dysregulation of enhancers can disrupt gene regulatory networks, culminating in aberrant transcriptional activity and tumor advancement [19, 23, 30, 31]. For example, an aberrantly activated enhancer cluster leads to the upregulation of the oncogene *SOX2* in breast and lung adenocarcinoma [32]. In estrogen receptor-positive (ER + ) breast cancer cells, the overexpression of FOXA1 instigates genome-wide enhancer reprogramming, which activates the hypoxia-inducible transcription factor HIF-2α and subsequently contributes to endocrine resistance and metastasis [33]. While comprehending the dynamics of cancer-associated enhancers is crucial, the specific molecular mechanisms underlying SMARCA4-driven enhancer reprogramming in LUAD remain inadequately characterized.

Here, we provide evidence that SMARCA4 activation in SMARCA4-deficient LUAD cells induces enhancer reprogramming and activates FOSL1-associated transcriptional programs that drive increased cell proliferation, migration, and tumor growth in our experimental models.

## Results

### SMARCA4 is upregulated in LUAD and its overexpression in SMARCA4-deficient LUAD cells enhances cell proliferation and migration

**Fig. 1:**
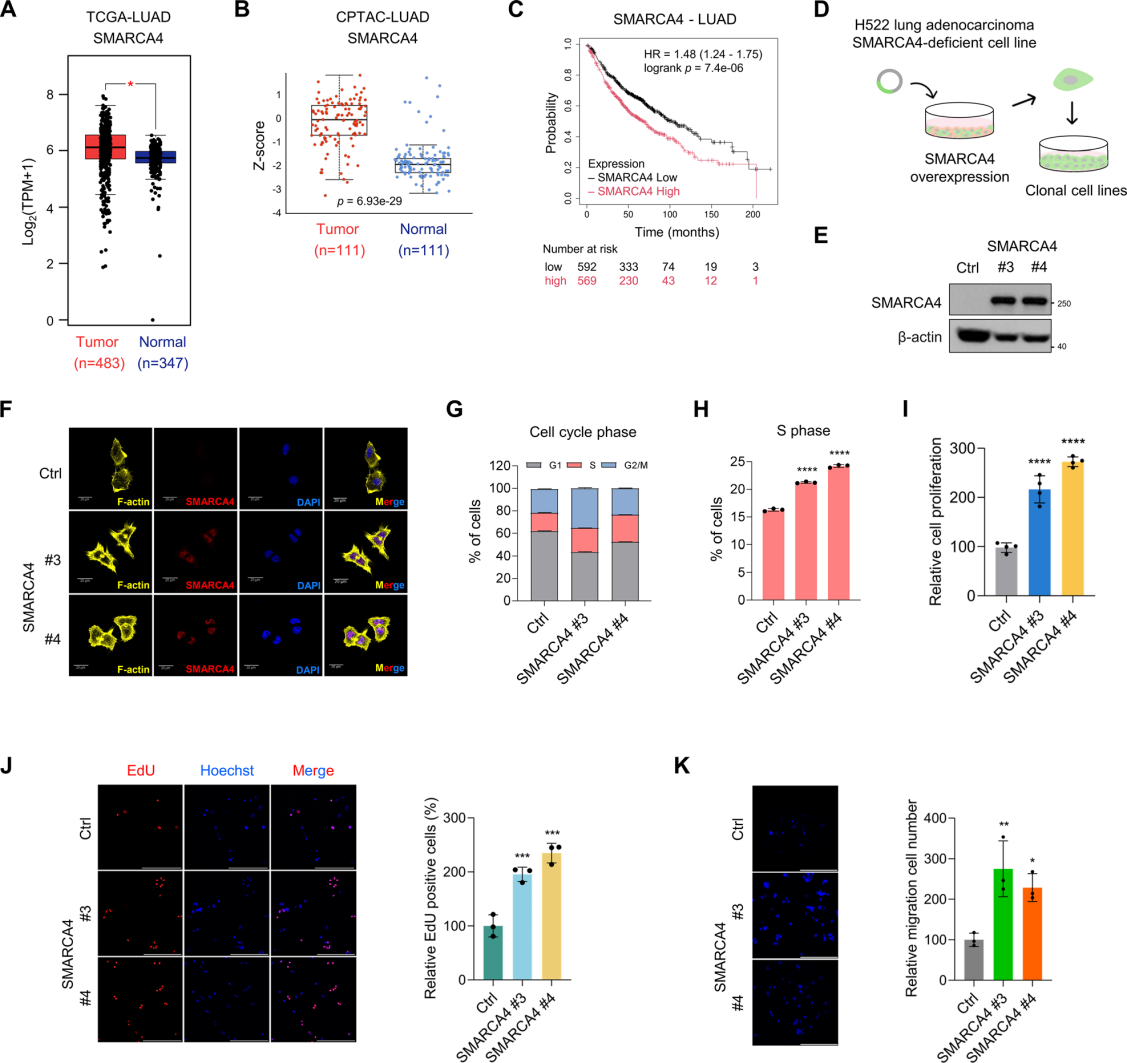
SMARCA4 is upregulated in LUAD and its overexpression in SMARCA4-deficient LUAD cells enhances cell proliferation and migration. **A** SMARCA4 expression levels in TCGA-LUAD tumors (red, *n* = 483) and matched normal tissues (blue, *n* = 347) from TCGA and GTEx using online tool, GEPIA2. *p < 0.05, *p* value cut off = 0.05. LUAD; lung adenocarcinoma. **B** SMARCA4 total protein levels of LUAD primary tumors (red, *n* = 111) and matched adjacent normal tissues (blue, *n* = 111) using UALCAN database from the CPTAC (Clinical Proteomic Tumor Analysis Consortium) dataset. *Z*-values represent standard deviations from the median across samples. **C** Overall survival of LUAD patients was evaluated using Kaplan-Meier plotter according to expression of SMARCA4. Probe set: SMARCA4 (214360_at). **D** Schematic of SMARCA4 overexpression and clonal cell lines generation. **E** Western blot analysis of SMARCA4 in H522 cells transfected with control (Ctrl) or SMARCA4 vector (two independent SMARCA4-expressing clones #3 and #4). β-actin was used as a loading control. **F** Immunofluorescence image of F-actin (yellow), SMARCA4 (red), DAPI (blue), and merged in Ctrl cells and two independent SMARCA4-expressing clones. Scale bar: 20 μm. **G** Percentage of H522 Ctrl cells and two independent SMARCA4-expressing clones in G1, S, and G2/M cell cycle phase. *n* = 3 biological replicates. **H** Percentage of H522 Ctrl cells and two independent SMARCA4-expressing clones in S phase. *n* = 3 biological replicates. **I** Relative cell viability of H522 Ctrl cells and two independent SMARCA4-expressing clones. Cell viability was determined using the ATP assay with CellTiter-Glo reagent. *n* = 4 biological replicates. **J** EdU assay of H522 Ctrl cells and two independent SMARCA4-expressing clones to detect cell proliferation. Representative fluorescent images of EdU and Hoechst (left) and the proportion of EdU-positive cells (right). Scale bar: 200 μm. *n* = 3 biological replicates. **K** Representative images (left) and quantification (right) of the migration abilities (stained with DAPI) for H522 Ctrl cells and two independent SMARCA4-expressing clones as measured by Transwell assay. Scale bar: 200 μm. *n* = 3 biological replicates. One-way ANOVA with Dunnett’s multiple comparisons test (**H**–**K**). Data are presented as the mean ± SD. *p < 0.05, **p < 0.01, ***p < 0.001, ****p < 0.0001.

To assess the expression profile of SMARCA4 in lung adenocarcinoma (LUAD), which represents the most common subtype of non-small cell lung cancer (NSCLC), we conducted an analysis of gene expression datasets obtained from The Cancer Genome Atlas (TCGA) and the Genotype-Tissue Expression (GTEx) databases. Notably, SMARCA4 mRNA levels were significantly elevated in LUAD tumor tissues compared to adjacent normal lung tissues (Fig. 1A). Consistently, proteomic data from the Clinical Proteomic Tumor Analysis Consortium (CPTAC) showed increased total SMARCA4 protein levels in LUAD tissues relative to normal tissues (Fig. 1B). We analyzed their expression profiles using the Kaplan-Meier plotter, which revealed that high SMARCA4 expression was associated with poor prognosis in patients with LUAD (Fig. 1C). These observations collectively suggest that SMARCA4 is highly expressed in LUAD, implicating a potential oncogenic function.

We employed the SMARCA4-deficient H522 LUAD cell line as a model system to examine the functional consequences of SMARCA4 overexpression in this experimental context. SMARCA4 was ectopically expressed in H522 cells, and stable SMARCA4-expressing clones were established through clonal expansion (Fig. 1D). The expression of SMARCA4 in individual clones was confirmed through western blot and immunofluorescence analyses (Fig. 1E, F). Importantly, flow cytometry analysis of cell cycle phase distribution revealed a significant increase in the S phase in SMARCA4-expressing clones compared to Ctrl (control) cells (Fig. 1G, H). The overexpression of SMARCA4 markedly enhanced cell viability when contrasted with Ctrl cells, as evidenced by elevated intracellular ATP levels (Fig. 1I). Edu incorporation assays indicated a substantial increase in the number of proliferating cells in SMARCA4-expressing clones (Fig. 1J). Furthermore, SMARCA4-expressing clones exhibited higher migratory capacity than Ctrl cells (Fig. 1K). These findings collectively indicate that SMARCA4 activity promotes cell proliferation and migration in our cellular model.

### SMARCA4 overexpression in SMARCA4-decifient LUAD cells reprograms epigenetic landscape and gene expression

**Fig. 2:**
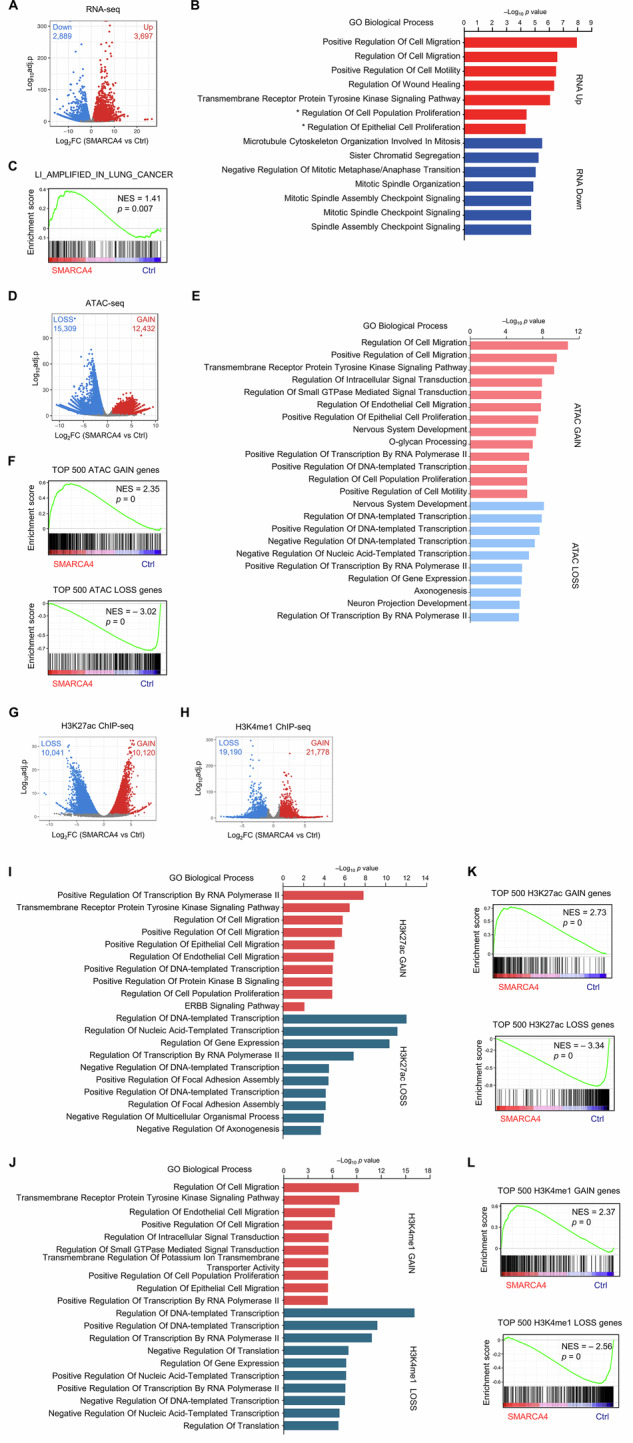
SMARCA4 overexpression in SMARCA4-deficient LUAD cells reprograms epigenetic landscape and gene expression. **A** A volcano plot showing differentially expressed genes between control (Ctrl) cells and two independent SMARCA4-expressing clones (#3 and #4), based on bulk RNA-sequencing. FDR < 0.05, |log_2_FC | > 1. *n* = 2 biological replicates (Ctrl: two independent experiments; SMARCA4: clones #3 and #4). **B** Top enriched Gene Ontology (GO) biological process terms for genes upregulated or downregulated upon SMARCA4 overexpression. GO Biological Process enrichment analysis was performed using Enrichr (GO Biological Process 2025 library). Enrichment significance was assessed using Fisher’s exact test, and GO terms were ranked based on Benjamini–Hochberg–adjusted *p* values. GO terms are ordered by enrichment rank, with the highest-ranked terms shown first. Selected lower-ranked proliferation-related terms (rank 29: regulation of cell population proliferation and rank 31: regulation of epithelial cell proliferation; marked with asterisks) are additionally indicated. **C** Gene set enrichment analysis (GSEA) of RNA-seq data from Ctrl cells and two independent SMARCA4-expressing clones showing greater upregulation of LUAD signature genes in SMARCA4 clones compared to Ctrl cells. (Gene set description: 183 genes are involved in increased copy numbers that correlate with increased expression across six LUAD cell lines). Normalized enrichment score (NES) and nominal *p* value were provided according to GSEA. **D** A volcano plot showing differentially accessible regions between Ctrl cells and SMARACA4-expressing clone #4. FDR < 0.05, |log_2_FC | > 1. *n* = 2 biological replicates. **E** Top enriched GO biological processes for the nearest genes of ATAC GAIN and LOSS regions upon SMARCA4 overexpression. **F** GSEA of RNA-seq data from Ctrl cells and two independent SMARCA4-expressing clones using the top 500 ATAC GAIN (top) or LOSS (bottom) genes. Normalized enrichment score (NES) and nominal *p* value were provided according to GSEA. **G** A volcano plot showing differential H3K27ac ChIP-seq peaks between Ctrl cells and SMARACA4-expressing clone #4. FDR < 0.05, |log_2_FC | > 1. *n* = 2 biological replicates. **H** A volcano plot showing differential H3K4me1 ChIP-seq peaks between Ctrl cells SMARACA4-expressing clone #4. FDR < 0.05, |log_2_FC | > 1. *n* = 2 biological replicates. **I** Top enriched GO biological processes for the nearest genes of H3K27ac GAIN and LOSS regions upon SMARCA4 overexpression. **J** Top enriched GO biological processes for the nearest genes of H3K4me1 GAIN and LOSS regions upon SMARCA4 overexpression. **K** GSEA of RNA-seq data from Ctrl cells and two independent SMARCA4-expressing clones using the top 500 H3K27ac GAIN (top) or LOSS (bottom) genes. Normalized enrichment score (NES) and nominal *p* value were provided according to GSEA. **L** GSEA of RNA-seq data from Ctrl cells and two independent SMARCA4-expressing clones using the top 500 H3K4me1 GAIN (left) or LOSS (right) genes. Normalized enrichment score (NES) and nominal *p* value were provided according to GSEA.

To evaluate the phenotypic changes in gene expression following SMARCA4 overexpression, we performed RNA-seq and identified 3697 upregulated and 2889 downregulated genes in SMARCA4-expressing clones compared to Ctrl cells (Fig. 2A, FDR < 0.05, |log_2_FC | > 1). Functional enrichment analysis of the differentially expressed genes revealed that genes upregulated upon SMARCA4 overexpression were significantly enriched in biological processes related to cell migration, motility and proliferation, consistent with the observed phenotypic changes (Fig. 2B). In contrast, the downregulated genes were found to be enriched in cell cycle checkpoint signaling pathways (Fig. 2B). To demonstrate the relevance of LUAD-associated oncogenic programs, we performed Gene Set Enrichment Analysis (GSEA) using the “LI_AMPLIFIED_IN_LUNG_CANCER” gene set, which comprises genes previously identified as amplified and overexpressed in LUAD [34]. Overexpression of SMARCA4 was significantly associated with enrichment of this gene set (Fig. 2C).

ATAC-seq revealed substantial changes in chromatin accessibility, with 12,432 regions exhibiting increased accessibility and 15,309 regions showing decreased accessibility following SMARCA4 overexpression, (Fig. 2D, FDR < 0.05, |log_2_FC | > 1). Genes associated with regions of increased accessibility were significantly enriched in pathways regulating cell migration, proliferation, and motility, whereas genes linked to decreased accessibility were associated with transcriptional regulation processes (Fig. 2E). GSEA showed that changes in chromatin accessibility induced by SMARCA4 overexpression are correlated with alterations in gene expression (Fig. 2F).

ChIP-seq analysis for two well-characterized enhancer marks, H3K27ac and H3K4me1 [24], was conducted to further investigate the alterations within the enhancer landscape following SMARCA4 overexpression. The differential peak analysis identified regions demonstrating both increased and decreased levels of H3K27ac and H3K4me1 (Fig. 2G, H, FDR < 0.05, |log_2_FC | > 1). Functional enrichment analysis of genes located near differential peaks revealed consistent patterns across the datasets generated from both H3K27ac and H3K4me1 ChIP-seq (Fig. 2I, J). Genes located near regions with elevated enhancer-associated histone marks were predominantly enriched in pathways relevant to cell proliferation and migration, while genes located near regions with diminished signals were associated with transcriptional regulation processes (Fig. 2I, J). Furthermore, GSEA demonstrated that the alterations in H3K27ac and H3K4me1 induced by ectopic expression of SMARCA4 correspond with changes in gene expression (Fig. 2K, L). Together, SMARCA4 activation in SMARCA4-deficient LUAD cells induces genome-wide alterations in epigenetic landscape and transcriptional programs. Notably, upregulated genes were frequently located near regions with increased chromatin accessibility and were associated with increased levels of active enhancer marks (H3K27ac and H3K4me1), further supporting their involvement in pathways related to cell proliferation and cell migration.

### SMARCA4 overexpression in SMARCA4-decifient LUAD cells results in SWI/SNF redistribution and regulates chromatin accessibility

To investigate the composition of SWI/SNF complexes before and after SMARCA4 overexpression, we performed co-immunoprecipitation (co-IP) of SMARCB1, a core subunit of the complex [13]. In the absence of SMARCA4, SMARCB1 maintained its interaction with SMARCC1, suggesting the formation of a residual SWI/SNF complex (Supplementary Fig. 1A). Upon ectopic expression of SMARCA4, SMARCB1 interacted with SMARCC1, SMARCA4 and SS18, indicating reassembly of a potentially functional SWI/SNF complex (Supplementary Fig. 1A). Co-IP experiments involving SMARCA4 in Ctrl cells did not yield any SWI/SNF subunits, thereby confirming the absence of SMARCA4 protein (Supplementary Fig. 1B). In contrast, in SMARCA4-expressing clones, SMARCA4 interacted with SMARCB1, SMARCC1, and SS18, supporting the assembly of a complete SWI/SNF complex (Supplementary Fig. 1B). This observation aligns with prior research [35] that established the essential role of SMARCA4 in the proper assembly of the SWI/SNF complex.

These findings suggest that although core components such as SMARCB1 and SMARCC1 can associate in the absence of SMARCA4, other subunits, including SS18 fail to incorporate. SMARCA4 facilitates the integration of these additional components, thereby promoting the formation of a functional SWI/SNF complex.

Given that SMARCB1 constitutes a component of the residual SWI/SNF complex in the absence of SMARCA4, we performed ChIP-seq analysis of SMARCB1 to examine its genome-wide binding profiles before and after SMARCA4 overexpression (Supplementary Fig. 1C). Differential peak analysis following SMARCA4 overexpression revealed 20,040 regions with increased SMARCB1 binding and 2873 regions with decreased binding (FDR < 0.05, |log_2_FC | > 1). Notably, SMARCB1 binding was significantly increased in SMARCA4-expressing clones compared to Ctrl cells (Supplementary Fig. 1C). Genes associated with increased SMARCB1 binding were highly enriched in cell proliferation and migration pathways, whereas genes associated with decreased SMARCB1 binding were enriched in transcription-related processes (Supplementary Fig. 1D).

To characterize the genomic localization of the reassembled SWI/SNF complex, we performed SMARCA4 ChIP-seq in SMARCA4-expressing clones. The chromatin occupancy of SMARCA4 was subsequently compared across regions classified as SMARCB1 GAIN, Retained, and LOSS (Supplementary Fig. 1E). Notably, regions with decreased SMARCB1 binding exhibited relatively weak SMARCA4 signals, whereas regions with increased SMARCB1 binding showed strong co-localization of SMARCA4 (Supplementary Fig. 1E). These findings suggest that SMARCA4 facilitates the redistribution of SWI/SNF complexes to specific chromatin regions upon SMARCA4 activation in SMARCA4-deficient LUAD cells.

Subsequently, we aimed to explore the functional implications of this redistribution concerning chromatin accessibility and histone modifications. Regions that exhibited a reduction in SMARCB1 binding showed a modest decrease in chromatin accessibility alongside diminished H3K27ac/H3K4me1 enrichment. In regions where SMARCB1 binding was retained, SMARCA4 exhibited partial co-localization; however, no significant changes were observed in chromatin accessibility or active enhancer marks (H3K27ac and H3K4me1) (Supplementary Fig. 1E). In contrast, regions with increased SMARCB1 binding demonstrated a pronounced increase in chromatin accessibility, as well as elevated levels of H3K27ac and H3K4me1 (Supplementary Fig. 1E). This was exemplified at the *ZNF468* and *SEMA3A* loci, respectively (Supplementary Fig. 1F). Furthermore, analysis of SWI/SNF occupancy in ATAC GAIN regions revealed that SMARCB1 and SMARCA4 co-localized at approximately 65% of these sites, representing a substantial proportion of chromatin regions with increased accessibility (Supplementary Fig. 1G). Together, these findings indicate that SMARCA4 incorporation into SWI/SNF complexes promotes chromatin opening at target sites, leading to enhanced chromatin accessibility and activation of distal regulatory elements, thereby reshaping chromatin architecture to modulate gene expression in our experimental LUAD model.

### SWI/SNF-mediated enhancer reprogramming activates oncogenic gene expression

**Fig. 3:**
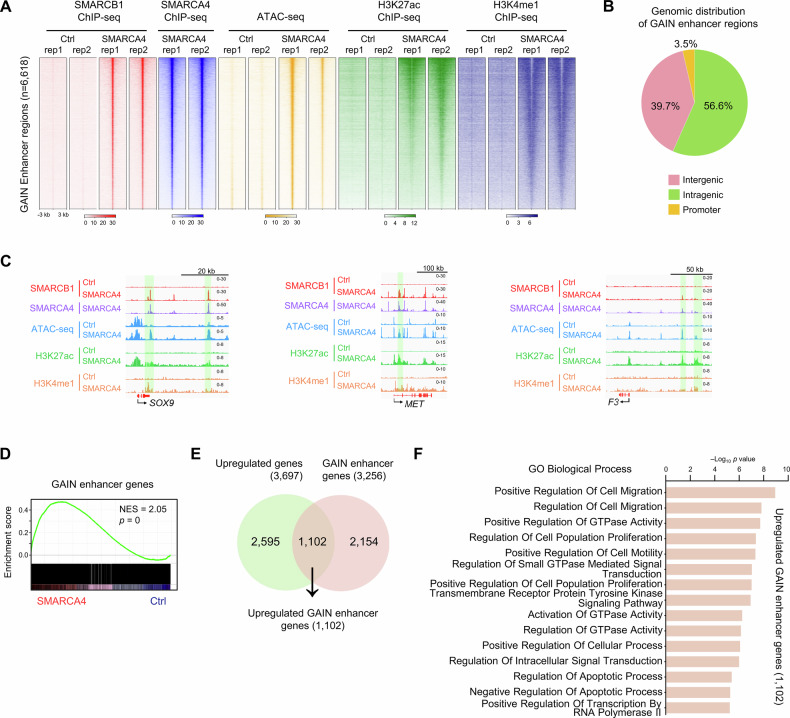
SWI/SNF-mediated enhancer reprogramming activates oncogenic gene expression. **A** Heatmaps of SMARCB1, SMARCA4, H3K27ac, H3K4me1 ChIP-seq signal and ATAC-seq signal in Ctrl cells and SMARACA4-expressing clone #4 at GAIN enhancer regions (*n* = 6618). *n* = 2 biological replicates. **B** Pie charts displaying the genomic distribution of GAIN enhancer regions. Intergenic; intergenic, Intragenic; exon, intron, transcription termination sites, Promoter; promoter. **C** IGV tracks of SMARCB1, SMARCA4, H3K27ac, and H3K4me1 enrichment (ChIP-seq) and chromatin accessibility (ATAC-seq) at the *SOX9, MET*, and *F3* loci in Ctrl and SMARACA4-expressing clone #4. **D** GSEA of RNA-seq data from Ctrl cells and two independent SMARCA4-expressing clones using a signature of GAIN enhancer genes. Normalized enrichment score (NES) and nominal *p* value were provided according to GSEA. **E** Venn diagram showing the overlapping status of upregulated genes (RNA Up) upon SMARCA4 overexpression and GAIN enhancer genes. **F** Top enriched GO biological processes for 1102 upregulated GAIN enhancer genes identified in Fig. 3E.

Given that ectopic expression of SMARCA4 induces the redistribution of the SWI/SNF complex and is correlated with augmented chromatin accessibility and the acquisition of active histone modifications (specifically H3K27ac and H3K4me1), we subsequently aimed to delineate enhancer elements that are newly activated under conditions of SMARCA4 overexpression. We identified a set of de novo activated regulatory regions with enhancer features, hereafter referred to as “GAIN enhancers” (*n* = 6618), characterized by newly acquired SMARCB1 and SMARCA4 binding, increased chromatin accessibility, and elevated levels of H3K27ac and H3K4me1 (Fig. 3A). These regions appeared to transition into an active enhancer state upon SMARCA4 overexpression, as they were previously inactive and lacked enhancer-associated features in SMARCA4-deficient Ctrl cells. These findings suggest that SMARCA4-containing SWI/SNF complexes play a role in enhancer activation through chromatin remodeling under conditions of SMARCA4 overexpression.

Genomic distribution analysis revealed that more than 97% of GAIN enhancer regions were localized in distal, exonic, and intronic regions, while only 3.5% were found in promoter regions (Fig. 3B). Ectopic expression of SMARCA4 led to a substantial increase in chromatin accessibility, accompanied by the enrichment of SMARCB1, H3K27ac, and H3K4me1 peaks that co-localized with SMARCA4 at the *SOX9*, *MET*, and *F3* loci (Fig. 3C).

GSEA demonstrated that genes associated with GAIN enhancers were significantly upregulated in SMARCA4-expressing clones compared to Ctrl cells (Fig. 3D). Moreover, 1102 genes associated with GAIN enhancer regions exhibited significant overlap with genes that were upregulated following SMARCA4 overexpression, thereby suggesting that these newly established enhancers facilitate the transcription of proximal target genes (Fig. 3E). Functional enrichment analysis revealed that GAIN enhancer-associated genes were strongly enriched in pathways related to cell proliferation, migration, and motility (Fig. 3F). Collectively, these findings support a model in which SMARCA4-containing SWI/SNF complexes promote de novo enhancer activation through chromatin remodeling, thereby driving transcriptional programs associated with increased cell proliferation and migration in this experimental setting.

### SMARCA4 overexpression in SMARCA4-deficient LUAD cells alters higher-order chromatin organization

Recent studies have demonstrated that the SWI/SNF complex contributes to the regulation of three-dimensional (3D) chromatin organization [36, 37]. To elucidate the role of SMARCA4 in higher-order chromatin structure, we performed high-throughput chromosome conformation capture (Hi-C) experiments in SMARCA4-deficient LUAD cells before and after SMARCA4 overexpression to identify genomic regions exhibiting significant changes in interaction strength. This analysis revealed differentially interacting regions (DIRs), comprising 3640 chromatin interactions that increased (DIR GAIN) and 3236 that decreased (DIR LOSS) (Supplementary Fig. 2A).

Genomic distribution analysis revealed that both DIR GAIN and DIR LOSS regions were predominantly enriched in intergenic or intragenic regions (99.4% and 96.6%, respectively), with only marginal proportions located in promoter regions (0.6% and 3.3%, respectively) (Supplementary Fig. 2B). To further characterize these regions, we integrated ATAC-seq, H3K27ac/H3K4me1, and SMARCB1 ChIP-seq datasets. DIR GAIN regions exhibited increased chromatin accessibility and were enriched for active enhancer marks and SMARCB1 binding, whereas DIR LOSS regions were associated with reduced accessibility and diminished active enhancer features (Supplementary Fig. 2C).

We subsequently explored whether these SMARCA4-induced 3D chromatin modifications are associated with transcriptional regulation. GSEA of RNA-seq data revealed that transcriptional changes were linked to alterations in chromatin interactions (Supplementary Fig. 2D). These findings suggest an association between SMARCA4 activity and alterations in higher-order chromatin organization, accompanied by changes in long-range chromatin interactions, within this experimental system. Consistent with these structural alterations, enhancer activation and increased chromatin accessibility were observed, as reflected by elevated levels of H3K27ac, H3K4me1, and SMARCB1 at DIR GAIN regions.

### FOSL1 is a cooperative transcription factor with SWI/SNF complex at GAIN enhancers

**Fig. 4:**
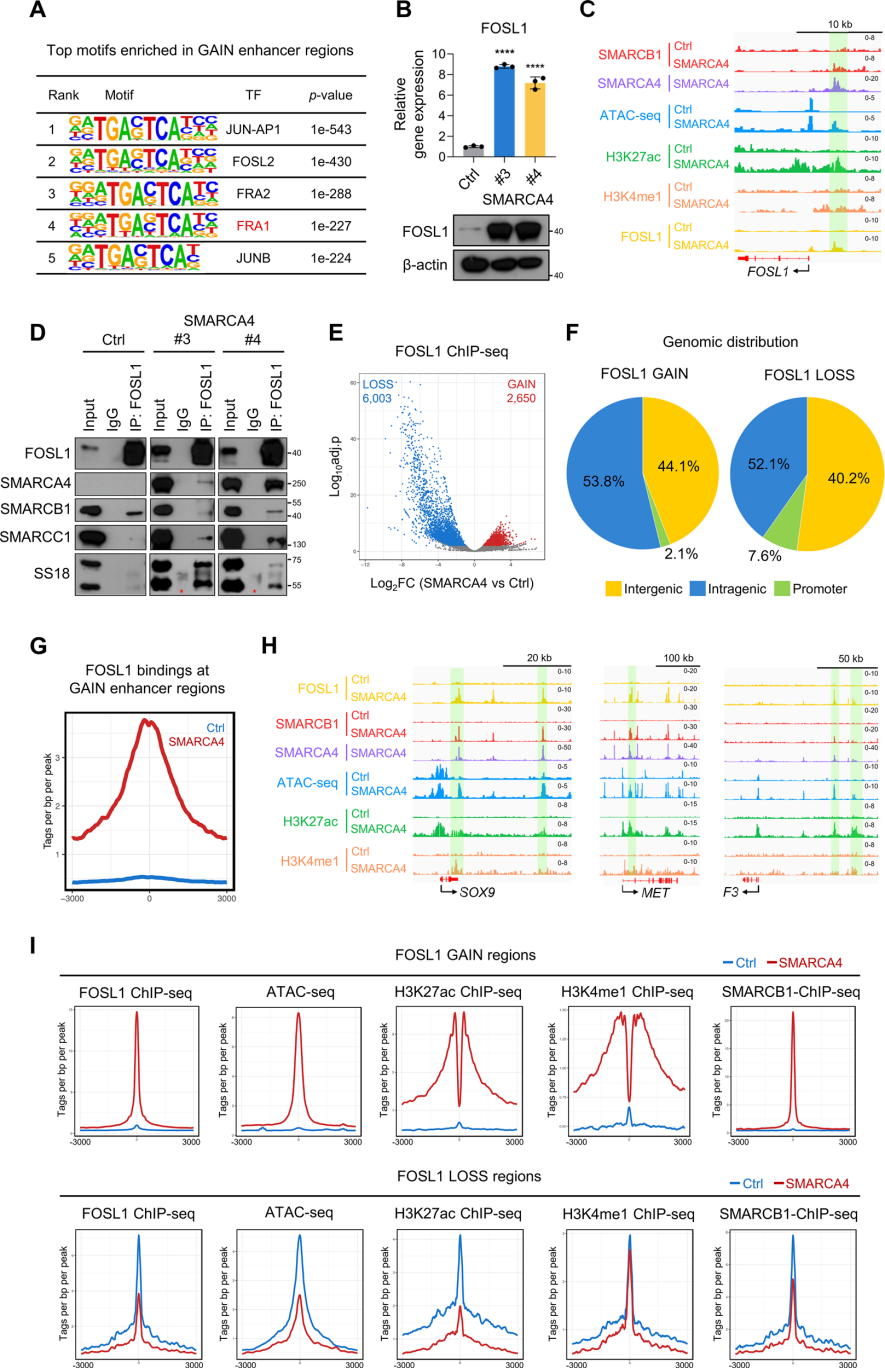
FOSL1 is a cooperative transcription factor with SWI/SNF complex at GAIN enhancers. **A** The motif analysis of GAIN enhancer regions identified by HOMER. **B** Normalized *FOSL1* mRNA expression was measured quantitative reverse transcription-polymerase chain reaction (RT-qPCR) in Ctrl cells and two independent SMARCA4-expressing clones. *n* = 3 biological replicates. (top) and western blot analysis of FOSL1 in Ctrl cells and SMARAC4 clones (bottom). β-actin was used as a loading control. **C** IGV tracks of SMARCB1, SMARCA4, H3K27ac, and H3K4me1 enrichment (ChIP-seq) and chromatin accessibility (ATAC-seq) at the *FOSL1* locus in Ctrl and SMARCA4-expressing clones. **D** Western blot analysis of FOSL1, major SWI/SNF core subunits (SMARCA4, SMARCB1, SMARCC1) and SS18 in Ctrl cells and two independent SMARCA4-expressing clones following immunoprecipitation of FOSL1. IgG bands are marked by red asterisks (*). **E** A volcano plot of differential FOSL1 ChIP-seq peaks between Ctrl cells and SMARCA4-expressing clones. FDR < 0.05, |log_2_FC | > 1. *n* = 2 biological replicates (Ctrl: two independent experiments; SMARCA4: clones #3 and #4). **F** Pie charts displaying genomic distribution of FOSL1 GAIN and LOSS regions. Intergenic; intergenic, Intragenic; exon, intron, transcription termination sites, Promoter; promoter. **G** Average plot of FOSL1 ChIP-seq signal in Ctrl cell and two independent SMARCA4-expressing clones at GAIN enhancer regions. **H** IGV tracks of FOSL1, SMARCB1, SMARCA4, H3K27ac, and H3K4me1 enrichment (ChIP-seq) and chromatin accessibility (ATAC-seq) at the *SOX9, MET*, and *F3* loci in Ctrl and SMARCA4-expressing clones. **I** Average plot of FOSL1, H3K27ac, H3K4me1, SMARCB1 ChIP-seq signal and ATAC-seq signal in Ctrl cell and SMARCA4-expressing clones at FOSL1 GAIN (top) and LOSS (bottom) regions. One-way ANOVA with Dunnett’s multiple comparisons test (**B**). Data are presented as the mean ± SD. ****p < 0.0001.

To identify TFs that cooperate with SMARCA4 to regulate GAIN enhancer activation and transcription, we performed motif analysis using Homer [38]. We found that the most significantly enriched motifs included binding sites for members of the AP-1 family (Fig. 4A). Among these, we focused on FOSL1 (also known as FRA1), as RNA-seq analysis showed that FOSL1 was upregulated in SMARCA4-expressing clones compared to Ctrl cells. Furthermore, FOSL1 protein levels were also elevated in SMARCA4-expressing cells (Fig. 4B). In addition, we identified a GAIN enhancer region at the FOSL1 locus, suggesting that FOSL1 may be upregulated by newly activated enhancers (Fig. 4C).

It has been well documented that AP-1 interacts with SWI/SNF complexes [37, 39]. To assess whether FOSL1 is associated with SWI/SNF complexes, we performed co-IP of FOSL1 followed by western blot analysis of SWI/SNF subunits (Fig. 4D). The results confirmed that FOSL1 interacts with partial SWI/SNF complexes in Ctrl cells, whereas in SMARCA4-expressing clones, FOSL1 associates with the fully assembled SWI/SNF complex (Fig. 4D). To evaluate whether FOSL1 binding was indeed increased at GAIN enhancer regions, we performed ChIP-seq of FOSL1 in both Ctrl cells and SMARCA4 clones (Fig. 4E). Differential binding analysis revealed that FOSL1 binding was increased at 2650 regions and decreased at 6,003 regions following SMARCA4 overexpression (FDR < 0.05, |log_2_FC | > 1). Analysis of peak distribution revealed that more than 90% of FOSL1 GAIN and LOSS regions were located outside promoter regions, confirming that FOSL1 predominantly binds to distal, exonic and intronic regions (Fig. 4F). In accordance with motif analysis, FOSL1 binding was highly enriched at GAIN enhancer regions in SMARCA4-expressing clones, as exemplified at the *SOX9*, *MET*, and *F3* loci (Fig. 4G, H).

Pioneer factors are TFs that can bind directly to closed chromatin and recruit additional TFs or histone-modifying enzymes to facilitate transcriptional activation [23, 25, 27]. To assess whether FOSL1 functions as a pioneer TF, we examined ChIP-seq signals for SMARCB1, H3K27ac, H3K4me1, as well as ATAC-seq signals, at FOSL1 GAIN and LOSS regions (Fig. 4I). In regions exhibiting enhanced FOSL1 binding, we observed increased chromatin accessibility and elevated SMARCB1 binding. Conversely, in regions with diminished FOSL1 binding, chromatin displayed reduced accessibility, accompanied by diminished SMARCB1 binding (Fig. 4I). This correlation between FOSL1 binding and SMARCB1 occupancy suggests that FOSL1 may be functionally associated with SWI/SNF complexes and contribute to chromatin remodeling. Moreover, concomitant changes in active enhancer marks (H3K27ac and H3K4me1) (Fig. 4I) are consistent with a role for FOSL1 in pioneer-like activity associated with enhancer activation following SMARCA4 overexpression.

### SMARCA4 inhibition attenuates oncogenic gene expression programs, leading to reduced cell proliferation and migration

**Fig. 5:**
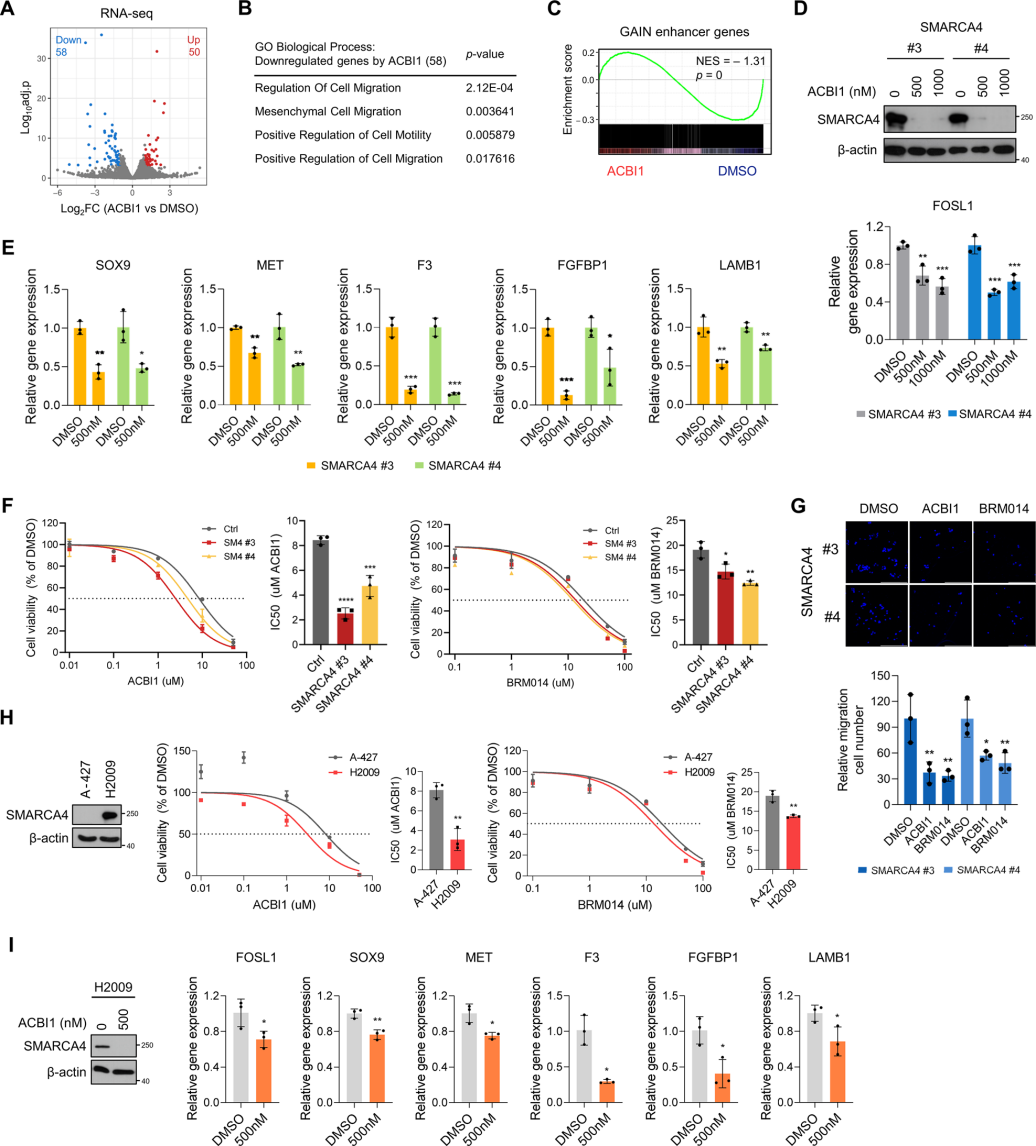
SMARCA4 inhibition attenuates oncogenic gene expression programs, leading to reduced cell proliferation and migration. **A** A volcano plot showing differentially expressed genes in SMARCA4-expressing clones treated with either ACBI1 (1 μM) or DMSO. FDR < 0.05, |log_2_FC | > 1. *n* = 2 biological replicates (two independent SMARCA4-expressing clones). **B** GO biological processes selected by migration- and motility-related functions for downregulated genes (RNA Down) upon ACBI1 treatment. **C** GSEA of RNA-seq data from SMARCA4-expressing clones treated with either ACBI1 (1 μM) or DMSO using a signature of GAIN enhancer genes. Normalized enrichment score (NES) and nominal *p* value were provided according to GSEA. **D** Western blot analysis of SMARCA4 in two independent SMARCA4-expressing clones treated with either ACBI1 at 500 and 1000 nM or DMSO. β-actin was used as a loading control (top). Normalized mRNA expression of *FOSL1* was measured by RT-qPCR in two independent clones expressing SMARCA4 treated either ACBI1 at 500 and 1000 nM or DMSO (bottom). *n* = 3 biological replicates. **E** Normalized mRNA expression levels of GAIN enhancer-associated genes—*SOX9, MET, F3, FGFBP1*, and *LAMB1*—were measured by RT-qPCR in two independent SMARCA4-expressing clones treated with either 500 nM ACBI1 or DMSO. *n* = 3 biological replicates. **F** Percentage of viable cells of Ctrl and two independent SMARCA4-expressing clones, measured using ATP assay with CellTiter-Glo reagents following treatment with increasing concentrations of ACBI1 for 3 days. The calculated IC50 values of ACBI1 are shown. *n* = 3 biological replicates. **G** Representative images (top) and quantification (bottom) of the migration experiment result (stained with DAPI) for two independent SMARCA4-expressing clones treated with DMSO, ACBI1 (5uM) or BRM014 (10uM) as measured by Transwell assay. Scale bar: 200 μm. *n* = 3 biological replicates. **H** Western blot analysis of SMARCA4 in A-427 (SMARCA4-deficient LUAD cell line) and H2009 (SMARCA4-proficient LUAD cell line). β-actin was used as a loading control. Percentage of viable cells of A-427 and H2009, measured using ATP assay with CellTiter-Glo reagents following treatment with increasing concentrations of ACBI1 for 3 days. The calculated IC50 values of ACBI1 are shown. *n* = 3 biological replicates. **I** Western blot analysis of SMARCA4 in H2009 cells treated with either ACBI1 at 500 or DMSO. β-actin was used as a loading control. Normalized mRNA expression of GAIN enhancer-associated genes including *FOSL1*, *SOX9*, *MET*, *F3*, *FGFBP1*, and *LAMB1* were measured by RT-qPCR in H2009 cells treated either ACBI1 at 500 nM or DMSO. *n* = 3 biological replicates. One-way ANOVA with Dunnett’s multiple comparisons test (**D**, **F**, **G**), Unpaired two-tailed Student’s *t* tests with Holm–Šidák correction for multiple comparisons (**E**). Data are presented as the mean ± SD. *p < 0.05, **p < 0.01, ***p < 0.001, ****p < 0.0001.

To assess global gene expression changes associated with SMARCA4 activity, RNA-seq analysis was performed following treatment with the PROTAC degrader ACBI1 [40], which specifically targets SWI/SNF ATPases (Fig. 5A, FDR < 0.05, |log₂FC | > 1). SMARCA4-induced transcriptional changes were negatively correlated with those observed upon ACBI1 treatment (Supplementary Fig. 3A, *R* = −0.15, *p* = 4.2e-32), indicating that the transcriptional program induced by SMARCA4 overexpression is maintained in a SMARCA4 activity-dependent manner and is broadly counteracted upon its inhibition. Functional enrichment analysis of downregulated genes upon ACBI1 treatment showed significant enrichment in pathways associated with cell migration and motility (Fig. 5B). Notably, treatment of SMARCA4-expressing clones with ACBI1 resulted in a significant reduction in the expression of GAIN enhancer-associated genes (Fig. 5C). Of note, FOSL1 mRNA expression was significantly decreased upon ACBI1 treatment, indicating that FOSL1 expression is regulated by SMARCA4 (Fig. 5D). Moreover, the expression levels of other representative GAIN enhancer-associated genes, including *SOX9*, *MET*, *F3*, *FGFBP1*, and *LAMB1*, were also significantly reduced as confirmed by RT-qPCR, supporting the role of SMARCA4 in maintaining their transcription (Fig. 5E).

To assess whether SMARCA4 inhibition induces anti-proliferative effects, we treated SMARCA4-expressing and Ctrl cells with ACBI1 (Fig. 5F). SMARCA4-expressing clones exhibited increased sensitivity to ACBI1 treatment compared to Ctrl cells, as evidenced by a reduction in IC50 values (Fig. 5F), supporting a dependency on SMARCA4 activity for cell growth. Similar results were observed with another SWI/SNF ATPases inhibitor, BRM014 [41] (Fig. 5F). Furthermore, both inhibitors significantly reduced cell migration (Fig. 5G).

Drug response was further assessed in two additional LUAD cell lines: A-427, which is SMARCA4-deficient, and H2009, which is SMARCA4-proficient (Fig. 5H). H2009 cells exhibited greater sensitivity to both ACBI1 and BRM014 compared to A-427 cells, reinforcing the notion that the anti-proliferative effects of ACBI1 and BRM014 are dependent on SMARCA4 activity (Fig. 5H). In addition to FOSL1, the mRNA expression levels of GAIN enhancer-associated genes, including *SOX9*, *MET*, *F3*, *FGFBP1*, and *LAMB1*, were also reduced upon ACBI1 treatment in H2009 cells, suggesting that SMARCA4 directly regulates the transcription of GAIN enhancer-associated genes (Fig. 5I). Collectively, these findings demonstrate that SMARCA4 inhibition suppresses genes associated with oncogenic processes and attenuates cell proliferation and migration in SMARCA4-expressing clones, highlighting the functional relevance of SMARCA4-dependent transcriptional regulation in this experimental context.

### SMARCA4 inhibition reduces chromatin accessibility and deactivates GAIN enhancers

**Fig. 6:**
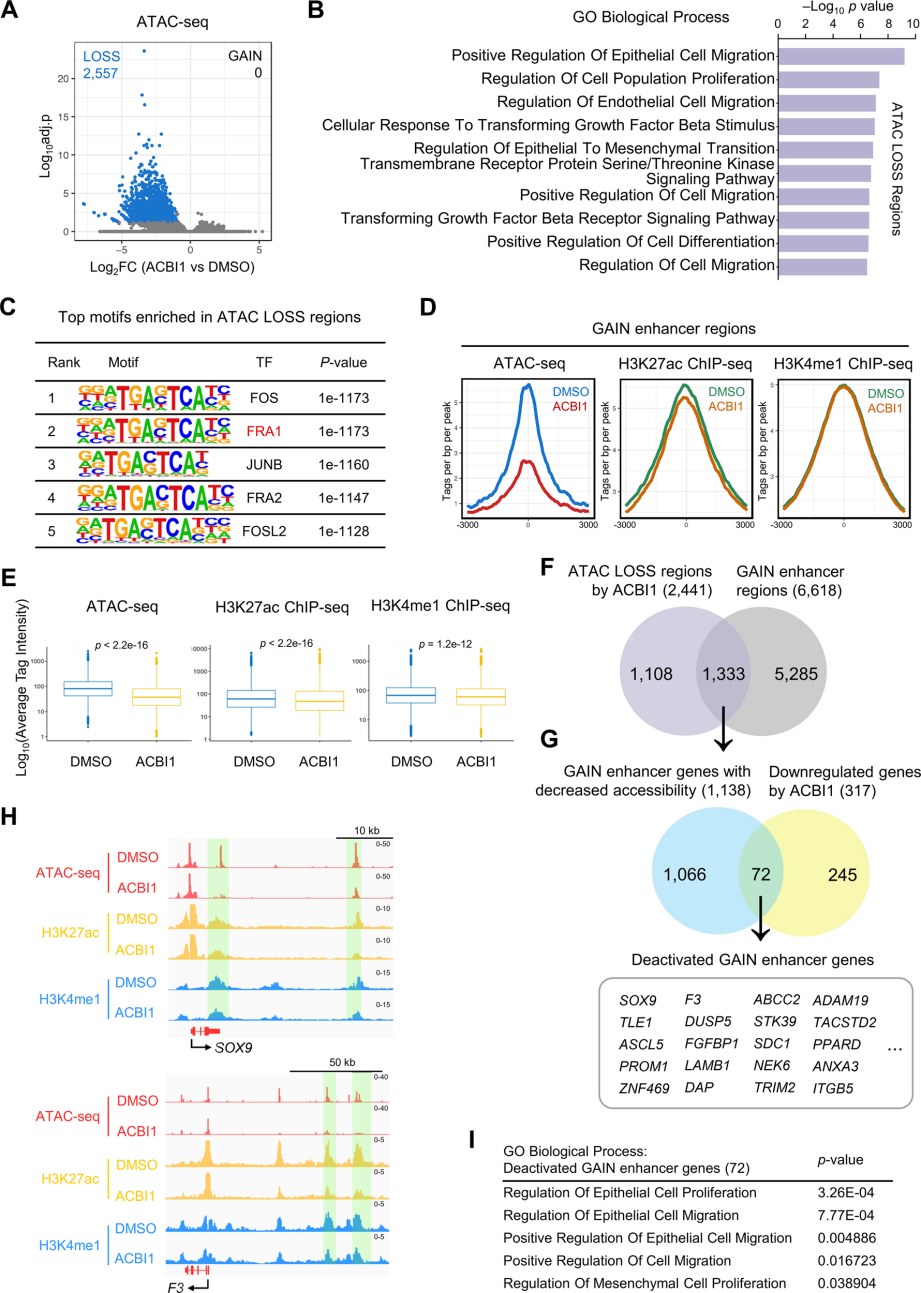
SMARCA4 inhibition reduces chromatin accessibility and deactivates GAIN enhancers. **A** A volcano plot showing differentially accessible regions in SMARCA4-expressing clones treated with either ACBI1 (1uM) or DMSO. Data from two independent clones were analyzed collectively by condition. FDR < 0.05, |log_2_FC | > 1. *n* = 2 biological replicates (two independent SMARCA4-expressing clones). **B** Top enriched GO biological processes for the nearest genes of ATAC LOSS regions upon ACBI1 treatment. **C** The motif analysis of ATAC LOSS regions identified by HOMER. **D** Average plots of ATAC-seq signal and H3K27ac, H3K4me1 ChIP-seq signal in SMARCA4-expressing clones treated with either ACBI1 (1uM) or DMSO at GAIN enhancer regions. *n* = 2 biological replicates (two independent SMARCA4-expressing clones). **E** Box plots showing the average ATAC-seq reads and H3K27ac and H3K4me1 ChIP-seq reads in SMARCA4-expressing clones treated with either ACBI1 (1uM) or DMSO. *p* values were calculated using the Wilcoxon rank-sum test. *n* = 2 biological replicates (two independent SMARCA4-expressing clones). **F** Venn diagram showing the overlapping status of ATAC LOSS regions upon ACBI1 treatment and GAIN enhancer regions. **G** Venn diagram showing the overlapping status of GAIN enhancer genes with decreased accessibility by ACBI1 treatment identified in Fig. 6F and downregulated genes (RNA Down) upon ACBI1 treatment. The genes that were overlapping were represented. **H** IGV tracks of H3K27ac and H3K4me1 enrichment (ChIP-seq) and chromatin accessibility (ATAC-seq) at the *SOX9* and *F3* loci in SMARCA4-expressing clones treated with either ACBI1 (1uM) or DMSO. **I** GO biological processes selected by proliferation-, migration-related functions for deactivated GAIN enhancer genes (*n* = 72) showing both accessibility reduction in ATAC-seq and downregulation in RNA-seq after ACBI1 treatment.

We next tested whether SMARCA4 directly affects chromatin accessibility at GAIN enhancers. ATAC-seq was performed following ACBI1 treatment, which revealed a significant reduction in chromatin accessibility at 2557 sites in SMARCA4-expressing clones (Fig. 6A, FDR < 0.05, |log_2_FC | > 1). Notably, no regions exhibiting increased accessibility were identified, indicating that the inhibition of SMARCA4 predominantly leads to chromatin condensation. Genes associated with decreased accessibility were enriched in pathways related to cell proliferation, migration, EMT, and cancer (Fig. 6B).

Motif analysis of sites with reduced accessibility following ACBI1 treatment identified an enrichment of FOSL1 motif, consistent with its presence in GAIN enhancer regions (Fig. 6C). This suggests that SMARCA4 regulates chromatin accessibility at loci containing the FOSL1 motif. A pronounced decline in chromatin accessibility was documented in GAIN enhancer regions, which was accompanied by a significant reduction in the levels of H3K27ac and a relatively lesser decrease in H3K4me1 (Fig. 6D, E).

Intriguingly, of the 2441 regions exhibiting reduced accessibility upon ACBI1 treatment, more than 54% corresponded to GAIN enhancer regions (Fig. 6F). By intersecting genes associated with GAIN enhancers that displayed diminished accessibility with genes that were downregulated following ACBI1 treatment, we identified a cohort of 72 genes potentially regulated by SMARCA4, representing a subset of GAIN enhancer targets that may undergo deactivation due to ACBI1 treatment (Fig. 6G). This was exemplified in the *SOX9* and *F3* loci (Fig. 6H). This subset of genes was significantly enriched in pathways related to cell proliferation and migration (Fig. 6I).

This gene set, which were upregulated upon SMARCA4 overexpression, exhibited a marked reduction in expression following ACBI1 treatment (Supplementary Fig. 3B). Moreover, SMARCA4 mRNA levels were positively correlated with the expression of 72 genes in the TCGA-LUAD dataset (Supplementary Fig. 3C, *R* = 0.2, *p* = 7.1e-06). Taken together, ACBI1-induced inhibition of SMARCA4 leads to the deactivation of GAIN enhancers by reducing chromatin accessibility and the enhancer marks deposition, accompanied by reduced expression of nearby genes. These findings underscore the functional relevance of SMARCA4 in modulating chromatin accessibility and gene expression in the cellular system examined in this study.

### FOSL1 is required for GAIN enhancer activation and its depletion reduces cell proliferation and migration

**Fig. 7:**
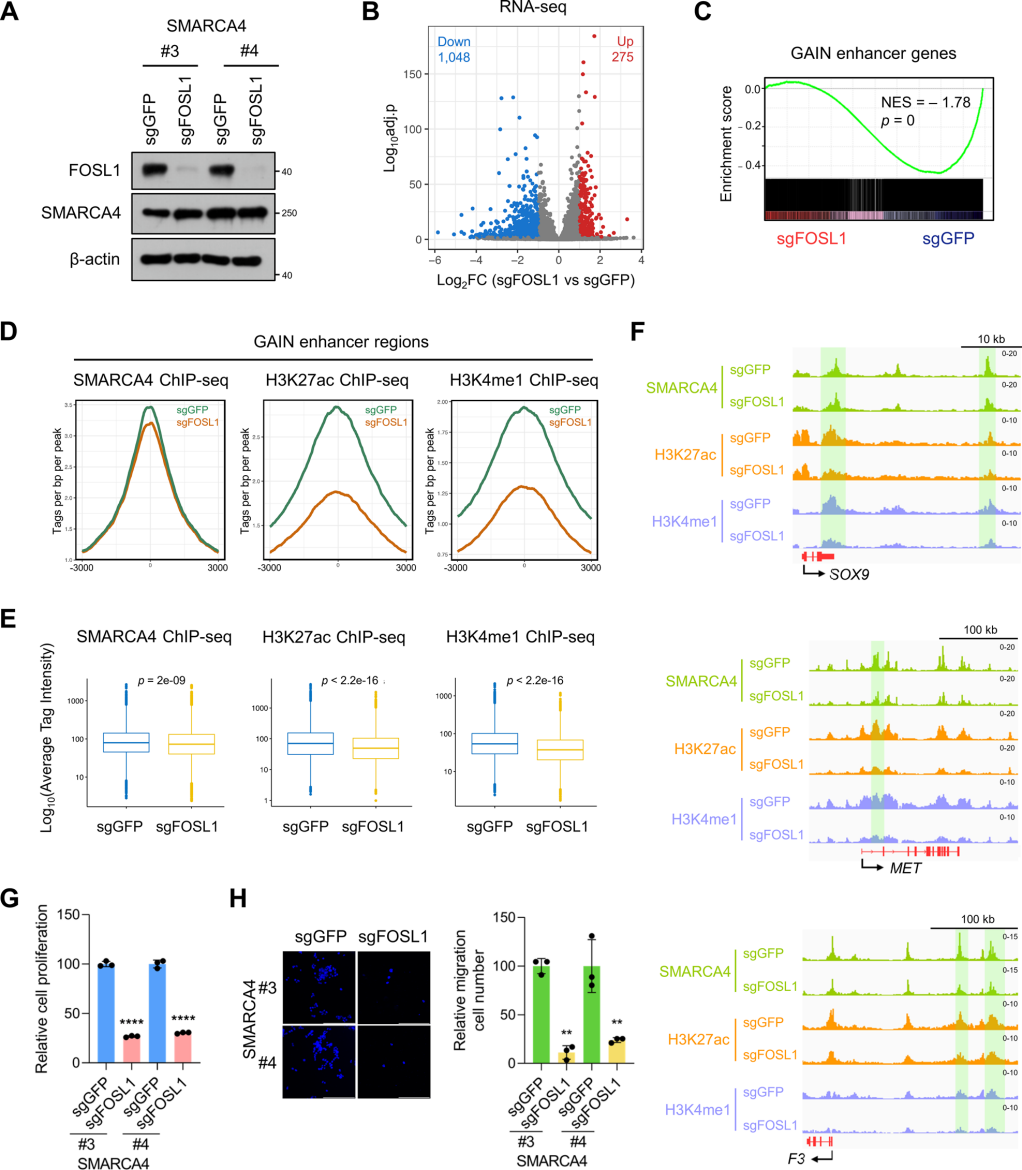
FOSL1 is required for GAIN enhancer activation and its depletion reduces cell proliferation and migration. **A** Western blot analysis of FOSL1 and SMARCA4 in two independent SMARCA4-expressing clones transduced with sgGFP (control) or sgFOSL1. β-actin was used as a loading control. **B** A volcano plot showing differentially expressed genes in SMARCA4-expressing clones transduced with sgGFP or sgFOSL1. FDR < 0.05, |log_2_FC | > 1. *n* = 4 biological replicates per condition (sgGFP or sgFOSL1), derived from two independent SMARCA4-expressing clones (#3 and #4; two replicates per clone). **C** GSEA of RNA-seq data from two independent SMARCA4-expressing clones transduced with sgGFP or sgFOSL1 using a signature of GAIN enhancer genes. Normalized enrichment score (NES) and nominal *p* values were provided according to GSEA. **D** Average plots of SMARCA4, H3K27ac and H3K4me1 ChIP-seq signal two independent SMARCA4-expressing clones transduced with sgGFP or sgFOSL1 at GAIN enhancer regions. *n* = 2 biological replicates (two independent SMARCA4-expressing clones). **E** Box plots showing the average SMARCA4, H3K27ac and H3K4me1 ChIP-seq reads in two independent SMARCA4-expressing clones transduced with sgGFP or sgFOSL1. *p* values were calculated using the Wilcoxon rank-sum test. *n* = 2 biological replicates (two independent SMARCA4-expressing clones). **F** IGV tracks of SMARCA4, H3K27ac and H3K4me1 enrichment (ChIP-seq) at the *SOX9*, *MET*, and *F3* loci in SMARCA4-expressing clones transduced with sgGFP or sgFOSL1. G Relative cell viability of two independent SMARCA4-expressing clones transduced with sgGFP or sgFOSL1. Cell viability was determined using the ATP assay with CellTiter-Glo reagent. *n* = 3 biological replicates. H Representative images (left) and quantification (right) of the migration experiment result (stained with DAPI) for two independent SMARCA4-expressing clones transduced with sgGFP or sgFOSL1 as measured by Transwell assay. Scale bar: 200 μm. *n* = 3 biological replicates. Unpaired two-tailed Student’s *t* tests with Holm–Šidák correction for multiple comparisons (**G**, **H**). Data are presented as the mean ± SD. **p < 0.01, ****p < 0.0001.

We have shown that FOSL1 potentially contributes to enhancer activation by regulating SWI/SNF recruitment and histone modifications (Fig. 4). Thus, to evaluate the necessity of FOSL1 in activating GAIN enhancers, we established CRISPR-mediated FOSL1 knockout in SMARCA4-expressing clones. Depletion of FOSL1 was confirmed, and SMARCA4 protein levels were unchanged in FOSL1-depleted SMARCA4 clones compared with FOSL1-proficient cells (Fig. 7A).

RNA-seq analysis revealed that FOSL1 depletion resulted in the upregulation of 275 genes and downregulation of 1048 genes (Fig. 7B, FDR < 0.05, |log_2_FC | > 1). SMARCA4-induced gene expression changes were negatively correlated with FOSL1 depletion-induced expression changes (Supplementary Fig. 4A, *R* = −0.18, *p* < 1.2e-44). The expression levels of the majority of GAIN enhancer-associated genes diminished following FOSL1 depletion (Fig. 7C). Furthermore, FOSL1 depletion resulted in a significant reduction in SMARCA4 binding as well as H3K27ac and H3K4me1 enrichment, as exemplified in the *SOX9*, *MET*, and *F3* loci (Fig. 7D–F). Changes in SMARCA4-induced H3K27ac and H3K4me1 enrichment were negatively correlated with those induced by FOSL1 depletion (Supplementary Fig. 4B, *R* = −0.4, *p* < 2.2e-16 and *R* = −0.24, *p* = <2.2e-16, respectively). These data suggest that FOSL1 facilitates SWI/SNF recruitment and histone modifications to activate GAIN enhancers.

The expression of 72 deactivated GAIN enhancer-associated genes identified in Fig. 6G was diminished following the depletion of FOSL1 (Supplementary Fig. 4C). Furthermore, the mRNA levels of FOSL1 exhibited a positive correlation with the expression of this gene set, as evidenced by the analysis of the TCGA-LUAD dataset (Supplementary Fig. 4D, *R* = 0.29, *p* = 1.5e-10). Notably, FOSL1 depletion decreased cell proliferation and migration (Fig. 7G, H). These data suggest that FOSL1 is associated with SWI/SNF occupancy and histone modifications at GAIN enhancers, potentially contributing to their activation.

### Impact of SMARCA4 and FOSL1 on tumor growth in vivo and clinical implications in LUAD

**Fig. 8:**
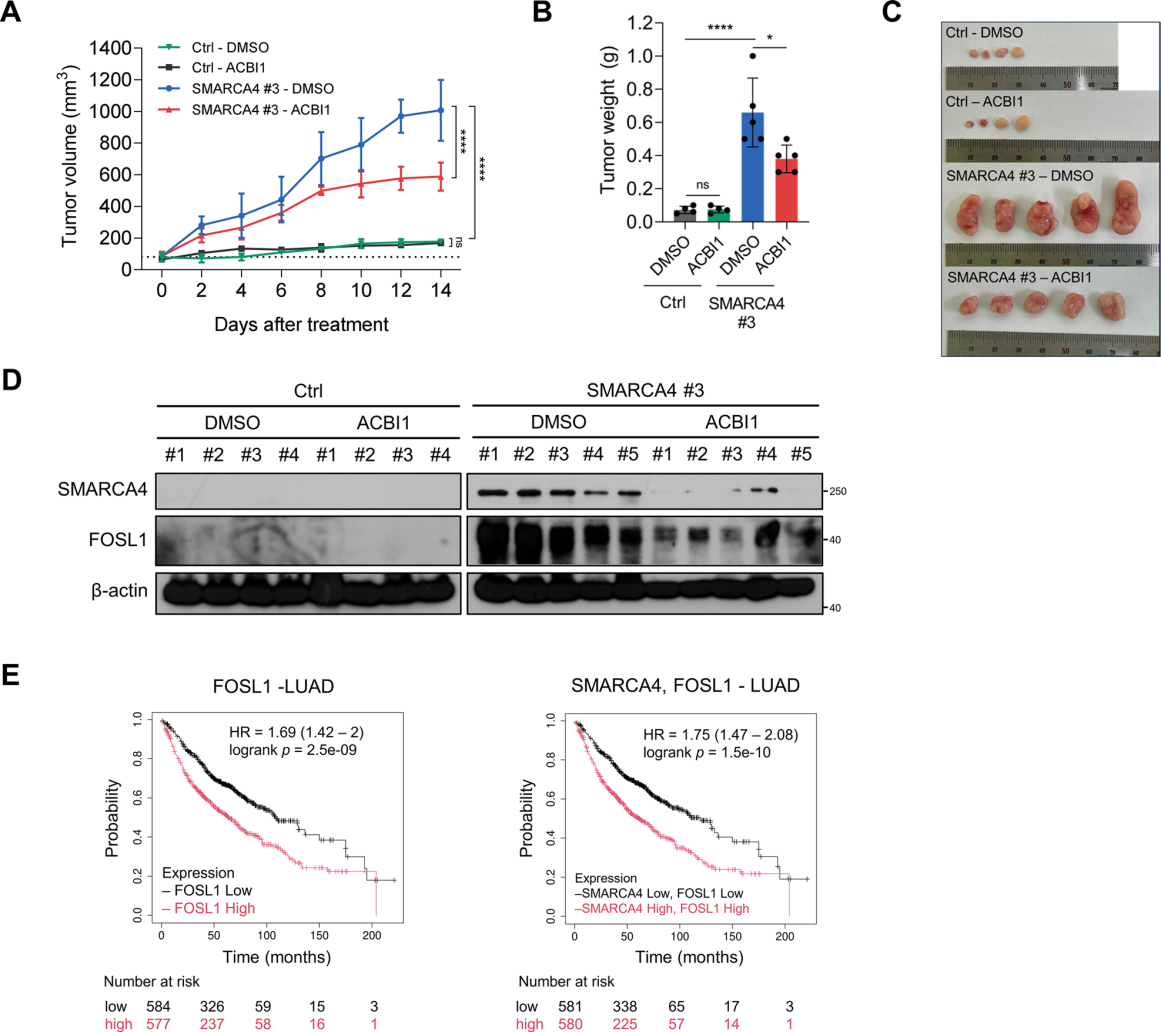
Impact of SMARCA4 and FOSL1 on tumor growth in vivo and clinical implications in LUAD. **A** Tumor volume of Ctrl cells or SMARCA4-expressing clone #3-derived xenograft tumors treated with either DMSO or ACBI1 (10 mg/kg). Ctrl group (*n* = 4), SMARCA4 group (*n* = 5). **B** Tumor weight of Ctrl cells or SMARCA4-expressing clone #3-derived xenograft tumors treated with either DMSO or ACBI1 (10 mg/kg). Ctrl group (*n* = 4), SMARCA4 group (*n* = 5). **C** Images showing xenografted tumors derived from Ctrl cells and SMARCA4-expressing clone #3 treated with either DMSO or ACBI1 (10 mg/kg). Ctrl group (*n* = 4), SMARCA4 group (*n* = 5). **D** Western blot analysis of SMARCA4 and FOSL1 in Ctrl cells or SMARCA4-expressing clone #3-derived xenograft tumors treated with either DMSO or ACBI1 (10 mg/kg). β-actin was used as a loading control. **E** Overall survival of LUAD patients was evaluated using Kaplan-Meier plotter according to expression of FOSL1 (left), SMARCA4 and FOSL1 (right). Probe set: SMARCA4 (214360_at), FOSL1 (204420_at). Two-way ANOVA with Tukey’s post hoc multiple comparisons test (**A**, **B**). Data from in vivo experiments are presented as the mean ± SEM for tumor growth and the mean ± SD for tumor weight. *p < 0.05, ****p < 0.0001, ns, not significant.

To evaluate the effects of SMARCA4 on tumor growth, we generated xenograft tumors using Ctrl cells and SMARCA4-expressing clone and assessed their response to ACBI1 treatment. Consistent with our in vitro findings, tumors in the SMARCA4 group exhibited greater growth and weight compared to those in the Ctrl group (Fig. 8A–C). Accordingly, ACBI1 treatment significantly suppressed tumor growth and reduced tumor weight in the SMARCA4 group compared to DMSO treatment. In contrast, ACBI1 treatment exhibited no substantial effect on tumor growth or weight in the Ctrl group.

We observed significantly elevated levels of FOSL1 expression in the SMARCA4 cohort as compared to the Ctrl cohort (Fig. 8D). Notably, FOSL1 expression was reduced upon ACBI1 treatment in the SMARCA4 group (Fig. 8D). Together, these results suggest that SMARCA4 overexpression is associated with enhanced tumorigenic phenotypes, potentially linked to increased FOSL1 expression in the context of our experimental model.

To determine the clinical implication of SMARCA4 or FOSL1, we analyzed their expression profiles using the Kaplan-Meier plotter. High expression levels of FOSL1 were associated with poor prognosis in patients with LUAD (Fig. 8E). Furthermore, based on the mean expression of SMARCA4 and FOSL1, samples were classified into two groups: SMARCA4 High, FOSL1 High and SMARCA4 Low, FOSL1 Low (Fig. 8E). Patients in SMARCA4 High, FOSL1 High group exhibited significantly worse overall survival compared to those in the SMARCA4 Low, FOSL1 Low group (Fig. 8E). Overall, these data suggest that SMARCA4 and FOSL1 expression levels are associated with overall survival LUAD patients.

## Discussion

Our findings reveal a context-dependent oncogenic function of SMARCA4 through epigenetic activation of enhancer programs in SMARCA4-deficient LUAD cellular models. Upon SMARCA4 overexpression, we identified de novo enhancer elements (GAIN enhancers) characterized by increased chromatin accessibility, SWI/SNF occupancy, and active histone modifications, indicating that SMARCA4-containing SWI/SNF complexes can convert repressed chromatin into functional regulatory elements. Notably, these enhancers were enriched near genes involved in proliferation and migration, supporting their role in tumor-associated transcriptional programs. Together, GAIN enhancer formation represents a key molecular signature of SMARCA4-mediated chromatin remodeling within our experimental system.

SMARCA4, the ATPase subunit of the SWI/SNF chromatin-remodeling complex, exerts a dual, context-dependent role in cancer as either a tumor suppressor or an oncogenic factor [17, 42, 43]. Its loss through mutation or deletion is associated with aggressive clinical features, elevated PD-L1 expression, and increased tumor mutational burden in NSCLC [44, 45]. Conversely, preserved or overexpressed SMARCA4 can promote tumor progression [14, 16], as exemplified by its role in sustaining the neuroendocrine phenotype in small cell lung cancer [46] and driving PRMT1-H4R3me2a-dependent transcriptional programs in colorectal cancer [14]. These findings underscore that SMARCA4 function is highly dependent on cofactor context, chromatin state, and epigenetic modifiers across tumor types.

We identified FOSL1 as a key transcription factor cooperating with SMARCA4–SWI/SNF to mediate enhancer activation. Consistent with pioneer-like activity, FOSL1 is associated with SWI/SNF occupancy and histone-modifying enzymes, including p300/CBP and MLL3/4 [29, 47, 48], promoting chromatin remodeling and deposition of active enhancer marks (H3K27ac and H3K4me1). This cooperation generates de novo GAIN enhancers that activate genes involved in proliferation and migration. Notably, a GAIN enhancer at the FOSL1 locus itself was associated with increased FOSL1 expression upon SMARCA4 overexpression, indicating a positive feedback loop that amplifies the enhancer-driven transcriptional network under the experimental conditions. Beyond FOSL1, motif analysis further revealed enrichment of additional AP-1 family members, including JUN, FOSL2, and JUNB, at GAIN enhancers (Fig. 4A), suggesting that cooperative and partially redundant AP-1 factors may contribute to enhancer activation [49, 50].

SWI/SNF–FOSL1-driven enhancer activation was correlated with transcription of nearby genes. GAIN-associated genes were enriched in pathways related to proliferation and migration and were upregulated upon SMARCA4 overexpression (Fig. 4). This gene set included genes previously implicated in tumor growth and metastasis, such *as SOX9, F3, LAMB1, FGFBP1, DUSP5*, and *TRIM2* [51–55]. Additional GAIN-linked genes, including *TLE1, PROM1, ABCC2* [56–61], and the NSCLC oncogene *MET* [62–64], were associated with poor prognosis in LUAD. Suppression of SMARCA4 activity or FOSL1 expression markedly reduced GAIN enhancer-associated gene expression, underscoring their transcriptional dependence on this regulatory interaction.

3D chromatin organization is critical for gene regulation and genome stability, and its disruption is a hallmark of tumorigenesis [65–68]. Hi-C analysis revealed that SMARCA4 overexpression in SMARCA4-deficient LUAD cells induces widespread alterations in long-range chromatin interactions, resulting in thousands of gained or lost differentially interacting regions (DIRs) (Supplementary Fig. 2). These structural changes were closely coupled with SMARCB1 binding, chromatin accessibility, and the enrichment of active histone marks, consistent with a role for SMARCA4 contributes to the modulating three-dimensional chromatin architecture and associated epigenetic and transcriptional states within this cellular system. However, how these structural and enhancer-level mechanisms converge to drive tumor-associated transcriptional programs will require further investigation.

Functional perturbation experiments further supported our model. FOSL1 depletion in SMARCA4-expressing clones markedly reduced SMARCA4 occupancy, chromatin accessibility, and active enhancer marks at GAIN regions (Fig. 7D), while pharmacological SMARCA4 inhibition produced similar but weaker effects (Fig. 6D). Disruption of either factor impaired proliferative and migratory phenotype, indicating that collapse of this enhancer-driven transcriptional program underlies the observed proliferative and migratory defects.

This study has several limitations. First, most mechanistic experiments were conducted using a single SMARCA4-deficient LUAD cell line (H522). While this system enables well-controlled analyses of SMARCA4-driven chromatin regulation, it does not fully capture the molecular and genetic heterogeneity of LUAD. Second, SMARCA4 was ectopically expressed, representing an artificial overexpression system rather than a physiological regulatory context. Accordingly, the extent of enhancer reprogramming observed here may not directly reflect SMARCA4 activity in patient tumors. Future studies using additional LUAD models and strategies to modulate endogenous SMARCA4 activity will be required to assess the generalizability of these findings. In addition, multi-omics sequencing analyses were performed with a limited number of biological replicates (*n* = 2). To address this limitation, we applied conservative analytical thresholds and focused on robust, high-confidence changes that were consistent across multiple independent genomic datasets, which were further supported by independent functional assays, collectively reinforcing the biological relevance of the identified regulatory interaction within this experimental context.

In summary, to interrogate the context-specific oncogenic function of SMARCA4, we established a SMARCA4-deficient LUAD cellular model with ectopic expression of SMARCA4 and examined its effects on chromatin structure and gene regulation using integrated epigenomic and transcriptional analyses. Within this experimental system, our analyses define a SMARCA4-mediated enhancer reprogramming mechanism in which SMARCA4 cooperates with FOSL1 to engage GAIN enhancers and activate tumor-associated transcriptional programs. Together, these findings suggest that SMARCA4 acts as a context-dependent epigenetic regulator with potential therapeutic relevance.

## Materials and methods

### Cell culture

The human non-small cell lung cancer (lung adenocarcinoma) cell line H522, A-427, and H2009 were obtained from the Korean Cell Line Bank. Cells were cultured in RPMI 1640 (LM011-01, WELGENE, Gyeongsan-si, Republic of Korea) supplemented with 10% fetal bovine serum (#S001-01, WELGENE) and gentamicin (10 μg/mL) at 37 °C in a 5% CO_2_-humidified atmosphere. Cell lines were authenticated by short tandem repeat (STR) profiling and were routinely assessed for mycoplasma contamination.

### Plasmids

GFP-SMARCA4 vector was a gift from Kyle Miller (Addgene plasmid #65391). For generating the control vector, the GFP-SMARCA4 vector was amplified using PCR with a PfuUltra high-fidelity DNA polymerase (#600380; Agilent) to remove the GFP-SMARCA4 sequence. All constructs were verified by Sanger sequencing. H522 cells were transfected with GFP-SMARCA4 or control vector using lipofectamine. Transfection medium was changed after 24 h of lipofectamine transfection. Cells stably expressing SMARCA4 proteins were established and maintained in medium with 5 ug/mL blasticidin (#15205; Sigma-Aldrich). For gene knockout, sgRNAs targeting FOSL1 (sgFOSL1) or the control (sgGFP) were cloned into the LentiCRISPRv2 vector (#52961; Addgene). Primer sequences are listed in Supplementary Table 1. Lentiviral vectors carrying single-guide RNA (sgRNA) against GFP or FOSL1 were produced in 293FT cells by transfecting plasmids with ViraPower lentiviral packaging mix (Thermo Fisher Scientific, Waltham, MA, USA) as described previously [69]. Retroviral vectors were transfected into 293FT cells using pUMVC (84490; Addgene, Watertown, MA, USA) and pCMV-VSV-G (#8454; Addgene). The viruses were harvested from the media after 48 h and transduced to target cells in 6 μg/mL of polybrene (#H9268; Sigma-Aldrich, St. Louis, MO, USA). After 24 h of incubation, the transduced cells were selected in 1 μg/mL of puromycin (#P8833; Sigma-Aldrich) for 6 d. Target gene knockout was validated using western blotting or RT-qPCR.

### Cell viability assay

A cell viability assay was performed using CellTiter-Glo 3D (#G9682; Promega, Madison, WI, USA), according to the manufacturer’s protocol. Cells (2 000–4 000 cells/well) were seeded in a 96-well plate. The next day, cells were treated with different doses of SMARCA2/4 dual inhibitors ACBI1 (#CS-0099076; Chemscene) and BRM014 (CS-0067946; Chemscene), and a DMSO (#D8418; Sigma-Aldrich) control.

### Transwell assay

1 × 10^4^ cells in 200 μl of cell suspension was loaded into the apical chamber Transwell (#3422; Corning), which was incubated together with 600 μl conditioned medium containing 10% FBS in the basolateral chamber and incubated at 37 °C for 24 h. The non-migratory cells on the apical chamber were removed with cotton balls. Migratory cells attached to the bottom of the membrane were fixed with 4% paraformaldehyde for 10 min at room temperature and were permeabilized in 0.25% Triton X-100 in PBS for 10 min at room temperature. Cells were washed three times with phosphate-buffered saline (PBS) at each step. Cells were labeled with DAPI (#D3571; Invitrogen) for 5 min at room temperature. After washing with PBS 2 times, slides were mounted with faramount mounting medium (#S3025, Dako). Images were acquired by a Leica confocal microscope and quantified using the ImageJ software.

### Immunofluorescence imaging

Cells (1 × 10^5^) were seeded on to sterile cover glasses coated with Poly-L-Lysine (#P4832, Sigma-Aldrich), and then placed in 6-well plate cell culture plates at 37 °C for 24 h, following which the wells were filled with 2 mL conditioned medium containing 10% FBS. Seeded cells on the cover glasses were fixed in 4% paraformaldehyde for 10 min at room temperature. Cells were washed 3 times with PBS, followed by permeabilization with 0.25% Triton X-100 in PBS for 10 min. After blocking with 4% bovine serum albumin (BSA) for 1 h at room temperature, cells were incubated with primary antibody SMARCA4 (# sc-17796; Santa Cruz) diluted in a 4% BSA in PBS overnight at 4 °C. After washing 3 times with PBS, cells were incubated with secondary antibodies Alexa Flour 647 (#A21235; Thermo Fisher Sceintific) and Rhodamine Phalloidin (#R415; Molecular Probes) for 1 h and washed 3 times with PBS. Cells were stained with DAPI (#D3571, Invitrogen) for 5 min at room temperature. Cells were washed 2 times and slides were mounted with faramount mounting medium (#S3025, Dako). Images were acquired by a Leica confocal microscope.

### Flow cytometry

Cells (1 × 10^5^) were fixed in cold 70% ethanol and incubated with RNase A (#10291021; Invirtogen in 0.1% BSA for 15 min at 37 °C. And cells were stained with propidium iodide. Stained cells were analyzed on FACSCanto II (BD Biosciences).

### EdU incorporation assay

Cell proliferation was evaluated using the Click-iT™ Plus EdU Alexa Fluor™ 594 Imaging Kit (#C10646; Invitrogen). All cells were treated with 10 μM EdU and processed according to the manufacturer’s staining protocol. Captured images were analyzed using ImageJ software. The number of EdU-positive cells was determined based on Hoechst nuclear staining and expressed as a percentage of the total number of cells per field.

### Protein extraction, Western blotting, and Co-IP assay

Cells were washed twice with PBS and collected. Whole cell lysate was obtained by incubating cells on ice for 30 min in cell lysis buffer (50 mM Tris-HCl [pH 7.5], 150 mM NaCl, 1% NP-40, 0.1% Na-deoxycholate, 50 mM NaF, 1 mM sodium pyrophosphate, 1 mM EDTA, and protease/phosphatase inhibitors), followed by centrifugation at 16,022 × *g* at 4 °C for 20 min. The supernatant was collected, and protein concentrations were quantified using a BCA assay (Thermo Fisher Scientific). Equal amounts of proteins were fractionated using 10% sodium dodecyl-polyacrylamide gel electrophoresis (SDS-PAGE) and transferred to nitrocellulose membranes (Cytiva, Marlborough, MA, USA). The membranes were blocked with 1× TBS-T (20 mM Tris-HCl [pH 8.0], 150 mM NaCl, and 0.1% Tween-20) containing 5% skim milk with agitation and immunoblotted with specific primary antibodies diluted in 1× TBS-T containing 5% skim milk at 4 °C overnight. The next day, the membranes were washed five times with 1× TBS-T and incubated with secondary antibodies for 1 h at 25 °C, followed by five washes. The proteins were then detected using an ECL kit (Thermo Fisher Scientific) and visualized with X-ray film (AGFA, Mortsel, Belgium). For the Co-IP assay, cell lysates were pre-cleared with 20 μl of Dynabeads (#10004D, #10002D; Invitrogen) for 4 h and incubated overnight with 20 μl Dynabeads and 5–10 μg antibodies or IgG at 4 °C in a rotor. Beads were then washed extensively with cell lysis buffer, and IP materials were eluted with SDS-PAGE loading dye and loaded onto SDS-PAGE gels.

### Antibodies

The following antibodies were used in this study: SMARCA4 (#ab110641; Abcam, Cambridge, UK; #sc-17796; Santa Cruz Biotechnology), FOSL1 (#AF4935; R&D Systems), β-Actin (#sc1616; Santa Cruz Biotechnology, Santa Cruz, CA, USA), SMARCB1 (#A301-087A; Bethyl Laboratories), SMARCC1 (#sc-32763; Santa Cruz Biotechnology), SS18 (#21792; Cell Signaling Technology), rabbit IgG (#ab37415; Abcam), mouse IgG (#ab37355; Abcam), goat IgG (#sc-2028; Santa Cruz Biotechnology).

### In vivo xenograft experiments

Xenograft tumors were generated in 4-week-old female BALB/c nude mice (Orient Bio, Seongnam-si, Republic of Korea) by subcutaneously injecting 2.0 × 10^6^ H522 control cells or SMARCA4-expressing cells suspended in 200 μl of 50% Matrigel (#356234; BD Bioscience) (*n* = 4–5 mice per group). One week after inoculation, DMSO or 10 mg/kg of ACBI1 (#CS-0099076; Chemscene) was administered every 2 days by intraperitoneal injections (IP), and the tumor volumes were calculated as follows: tumor volume (mm^3^) = 1/2(length × width^2^). Sample size was not predetermined using statistical methods. The number of animals used was based on prior experience and previously published studies. No animals or samples were excluded from the analysis, and no pre-established inclusion or exclusion criteria were applied. Animals were assigned to experimental groups based on genotype, and no method of randomization was used. Investigators were not blinded to group allocation during the experiments or outcome assessment.

### RNA-seq, data processing, and analysis

Bulk RNA-seq was performed on Ctrl (control) cells and two independent SMARCA4-expressing clonal cell lines. RNA was extracted using TRI Reagent (#TR-118; Molecular Research Center, Cincinnati, OH, USA) according to the manufacturer’s instructions. Sequencing libraries were generated according to the standard protocol of Illumina (San Diego, CA, USA) for high-throughput sequencing. The transcriptome was then sequenced using a Genome Analyzer IIx (Illumina) as previously described [70]. Paired-end RNA-seq libraries were generated, and sequencing reads were trimmed using Trimmomatic to remove adapter sequences and low-quality bases. Trimmed reads were aligned to the human reference genome (hg19) using STAR with default alignment parameters. Aligned BAM files were converted into HOMER tag directories using makeTagDirectory for downstream analyses. Gene-level raw read counts were quantified using analyzeRepeats.pl in HOMER with exon-based counting. Differential expression analysis was performed using raw count matrices, with biological replicates treated as independent samples in the statistical model. Differentially expressed genes (DEGs) were identified using the HOMER script getDiffExpression.pl, which applies library-size normalization and replicate-aware statistical testing. Statistical significance was determined using false discovery rate (FDR) correction based on the Benjamini–Hochberg method, and genes with an FDR < 0.05 and an absolute log_2_ fold change (|log_2_FC | > 1) were considered differentially expressed.

### RT-qPCR analysis

Total RNA (2 μg) was reverse transcribed for cDNA synthesis, and real-time qPCR analysis was performed as previously described [71]. Primer sequences used for RT-qPCR are listed in Supplementary Table 2.

### ATAC-seq, data processing, and analysis

ATAC-seq was performed as previously described [72, 73]. Fifty thousand cells were lysed with 50 μl of RSB buffer (10 mM Tris-Cl [pH 7.4], 10 mM NaCl, and 3 mM MgCl_2_) containing 0.1% NP-40, 0.1% Tween-20, and 0.01% digitonin and incubated for 5 min at 4 °C. After lysis, 1 mL of cold RSB buffer containing 0.1% Tween-20 was added, and the tubes were inverted thrice to mix. After centrifuging at 379 × *g* for 10 min, nuclei were resuspended with 50 μl of Illumina 1X TD buffer containing 2.5 μl of Tn5 Transposase and incubated for 30 min at 37 °C in a thermomixer at 68 × *g*. DNA was purified using a QIAGEN MinElute PCR purification kit and then amplified with Nextera sequencing primers and NEBNext high-fidelity 2X PCR master mix for 15 cycles. Purified PCR products were deep-sequenced (paired-end 2 × 75 bp) using NextSeq500 (Illumina). Paired-end ATAC-seq reads were trimmed using Trimmomatic to remove adapter sequences and low-quality bases, and aligned to the human reference genome (hg19) using Bowtie2. Aligned reads were filtered to remove mitochondrial reads and PCR duplicates using SAMtools and Picard. Processed BAM files were converted into HOMER tag directories using makeTagDirectory, which served as the unified input format for downstream ATAC-seq analyses. Tag directories were generated independently for each biological replicate. Accessible chromatin regions were identified by peak calling on each replicate using the findPeaks function in HOMER with the -style factor parameter. Peaks from all samples were merged to generate a common set of accessible regions for comparative analyses. Peak annotation and normalized peak intensity quantification were performed using annotatePeaks.pl, which computes library-size–normalized tag counts for each peak region. For differential peak analysis, peaks were merged across samples to generate a common peak set. Peak annotation and normalized peak intensity quantification were performed using annotatePeaks.pl. Differential chromatin accessibility analysis was performed using HOMER getDiffExpression.pl with replicate-aware statistical testing. Peaks with an FDR < 0.05 and an absolute log_2_ fold change (|log_2_FC | > 1) were considered significantly different. For IGV visualization, ATAC-seq signal tracks were generated using makeUCSCfile with the -style chipseq option. For heatmap-based visualization, these signal tracks were converted into bigWig format, and signal intensities over genomic regions of interest were extracted using computeMatrix, followed by visualization using plotHeatmap. Motif enrichment analysis was conducted using findMotifsGenome.pl with the -size given parameter to identify transcription factor binding motifs enriched within ATAC-seq peaks.

### ChIP-seq, data processing, and analysis

Cells (2 × 10^7^) were crosslinked with 1% formaldehyde for 20 min at 25 °C. The reaction was terminated by adding 0.125 M glycine for 5 min. Cells were then lysed with 1 mL of cell lysis buffer (10 mM Tris-Cl [pH 8.0], 10 mM NaCl, and 0.2% NP-40) with protease inhibitors and incubated for 30 min at 4 °C in a rotor. Nuclei were isolated via centrifugation at 853 × *g* for 5 min. Pellets were resuspended with 500 μl of nuclei lysis buffer (50 mM Tris-Cl [pH 8.0], 10 mM EDTA, and 1% SDS) with protease inhibitors followed by incubation for 15 min at 4 °C. Lysed chromatins were digested using MNase (#LS004797; Worthington Biological Corporation, Lakewood, NJ, USA) for 20 min and sheared to an average chromatin fragment size of 200–400 bp using a QSonica’s Q500 sonicator. Sonicated chromatins were diluted with 2.5 mL of IP dilution buffer (20 mM Tris-Cl [pH 8.0], 150 mM NaCl, 2 mM EDTA, 0.01% SDS, and 1% Triton X-100) and then incubated with 5–10 μg of antibodies and 50 μl of Protein A or G Dynabeads overnight at 4 °C in a rotor. Immunoprecipitated beads were washed twice with low salt concentration wash buffer (20 mM Tris-Cl [pH 8.0], 150 mM NaCl, 2 mM EDTA, 0.1% SDS, and 1% Triton X-100), once with high salt concentration wash buffer (20 mM Tris-Cl [pH 8.0], 500 mM NaCl, 2 mM EDTA, 0.1% SDS, and 1% Triton X-100), once with final LiCl wash buffer (10 mM Tris-Cl [pH 8.0], 0.25 M LiCl, 1 mM EDTA, 1% NP-40, and 1% Na-deoxycholate), and twice with 1X TE (10 mM Tris-Cl [pH 8.0] and 1 mM EDTA). Chromatins were then eluted with 300 μl of elution buffer (100 mM sodium bicarbonate and 1% SDS). RNase A (#10291021; Invirtogen) and 0.25 M NaCl were added, and the chromatins were incubated overnight at 65 °C to reverse crosslinks. The next day, 100 μg of Proteinase K (Invitrogen) was added and incubated at 65 °C for 4 h, extracted using phenol:chloroform:isoamyl alcohol (#P2069; Sigma-Aldrich), and eluted in 50 μl of 1X TE. ChIP DNA and input DNA libraries were prepared using the NEBNext Ultra II DNA Library Prep Kit (#E7645; NEB) according to the manufacturer’s instructions. Single-end ChIP-seq libraries were generated for H3K27ac, H3K4me1, SMARCB1, SMARCA4, and FOSL1. Sequencing reads were trimmed using Trimmomatic and aligned to the human reference genome (hg19) using Bowtie2 with the —very-sensitive option. Aligned reads were filtered to remove mitochondrial reads and PCR duplicates using SAMtools and Picard. Processed BAM files were converted into HOMER tag directories using makeTagDirectory, which were generated independently for each biological replicate. Peak calling was performed using HOMER findPeaks with matched input samples as controls. ChIP-seq datasets (SMARCB1, SMARCA4, and FOSL1) were analyzed using the -style factor parameter, whereas histone modification datasets (H3K27ac and H3K4me1) were analyzed using the -style histone parameter. For differential peak analysis, peaks were merged across samples to generate a common peak set. Peak annotation and normalized peak intensity quantification were performed using annotatePeaks.pl. Differential binding analysis was performed using HOMER getDiffExpression.pl with replicate-aware statistical testing. Peaks with an FDR < 0.05 and an absolute log_2_ fold change (|log_2_FC | > 1) were considered significantly different between conditions. For IGV visualization, ChIP-seq signal tracks were generated using makeUCSCfile with the -style chipseq option. For heatmap-based visualization, these signal tracks were converted into bigWig format, and signal intensities over genomic regions of interest were extracted using computeMatrix, followed by visualization using plotHeatmap. Motif enrichment analysis was conducted using findMotifsGenome.pl with the -size given parameter.

### Hi-C, data processing, and analysis

Hi-C was performed in duplicate (two Ctrl replicates from independent cultures, and one replicate from each of two independent SMARCA4-expressing clones #3 and #4). 1 × 10^6^ cells were crosslinked with 0.3 M DSG at RT for 10 min and 1% formaldehyde at RT for 10 min. Hi-C samples were processed using the Dovetail® TopoLink™ Kit (#21010; Dovetail Genomics) according to the manufacturer’s protocol and sequenced with AVITI platform. Hi-C reads were aligned to the human reference genome (hg19) using BWA-MEM with the -SP5M option. Aligned reads were converted to BAM format using SAMtools, and valid Hi-C contact pairs were extracted using the pairtools suite. PCR duplicates were marked, and only valid interaction pairs were retained for downstream analyses. Contact matrices were generated at 1 kb and 500 kb resolutions using cooler cload pairix and subsequently balanced using iterative correction (cooler balance). Pixel-level interaction data were extracted using cooler dump -t pixels —join. Replicates within each condition were merged and averaged at the pixel level. Only intra-chromosomal interactions were retained for differential interaction analysis. Normalization and differential interaction analysis between control and SMARCA4-expressing cells were performed using the HiCcompare R package with LOESS normalization (hic_loess). Statistically significant differentially interacting regions (DIRs) were identified using hic_compare with an adjusted *p* value threshold of <0.05. DIRs were annotated by overlapping with gene annotations from TxDb.Hsapiens.UCSC.hg19.knownGene. Volcano plots were generated to visualize differences in interaction frequencies.

### Functional enrichment analysis

To understand the regulatory programs and functional implications associated with SMARCA4 activity, we performed functional enrichment analysis on gene sets derived from RNA-seq, ChIP-seq, and ATAC-seq analyses using Enrichr (https://maayanlab.cloud/Enrichr/) [74–76]. Gene Ontology (GO) Biological Process enrichment was assessed using Fisher’s exact test, with *p* values adjusted for multiple testing using the Benjamini–Hochberg method. GO terms were ranked according to their adjusted *p* values, with lower adjusted *p* values corresponding to higher enrichment ranks. Bar graphs display enriched GO terms in descending order starting from the top-ranked terms, and representative enriched pathways were selected for visualization.

### Analysis of public datasets

Analysis of TCGA and GTEx datasets were performed with GFPIA2 (http://gepia2.cancer-pku.cn/#analysis). Analysis of clinical proteomic tumor analysis consortium (CPTAC) dataset was performed with UALCAN portal (https://ualcan.path.uab.edu/cgi-bin/ualcan-res-prot.pl). Overall survival analysis was performed with Kaplan-Meier Plotter (https://kmplot.com/analysis/).

### Statistical analysis

All statistical analyses were performed using GraphPad Prism version 8 (GraphPad Software, Boston, MA, USA). Data normality was assessed using the Shapiro–Wilk test, and equality of variances between groups was evaluated using the F-test. For comparisons between two groups, statistical significance was assessed using an unpaired two-tailed Student’s *t* test. For experiments in which more than two experimental groups were compared against a single shared control, one-way analysis of variance (ANOVA) followed by Dunnett’s multiple comparisons test was applied. This approach was used both for comparisons between control cells and multiple independent SMARCA4-expressing clones, and for within-clone analyses in which multiple experimental conditions (e.g., different drug concentrations) were compared against a common control condition (DMSO) within the same clone. In experiments where control and experimental conditions were compared independently within individual SMARCA4-expressing clones (#3 and #4), and each comparison involved only two conditions (e.g., DMSO versus a single drug concentration, or sgGFP versus sgFOSL1), unpaired two-tailed Student’s *t* tests were performed within each clone. When multiple such within-clone comparisons were presented within the same figure, *p* values were adjusted using the Holm–Šidák method to account for multiple testing. All quantitative data are presented as mean ± standard deviation (SD). A *p* value < 0.05 was considered statistically significant. The number of biological replicates and the statistical tests used for each experiment are specified in the corresponding figure legends. No statistical methods were used to predetermine sample size.

### Illustration tools

The schematic illustration of graphical abstract was created with BioRender (https://www.biorender.com/).

## Supplementary information

Supplementary figures and legends

Supplementary Tables

Original western blot

## Data Availability

All the sequencing data generated in this study can be accessed through the European Nucleotide Archive (ENA) using the project accession code PRJEB89867.
