# Supplementary material for: SMARCA4 activation engages FOSL1 to drive enhancer reprogramming and tumorigenic phenotypes in SMARCA4-deficient LUAD cells
Source: Cell Death Discov. 2026 Apr 20;12:262. doi: 10.1038/s41420-026-03100-3 (PMC13223256; doi:10.1038/s41420-026-03100-3)
Supplement: Supplementary file 1 — Supplementary figures and legends [file 41420_2026_3100_MOESM1_ESM.docx]

**Supplementary Fig 1. SMARCA4 overexpression in SMARCA4-deficient LUAD cells results in SWI/SNF redistribution and regulates chromatin accessibility**

**
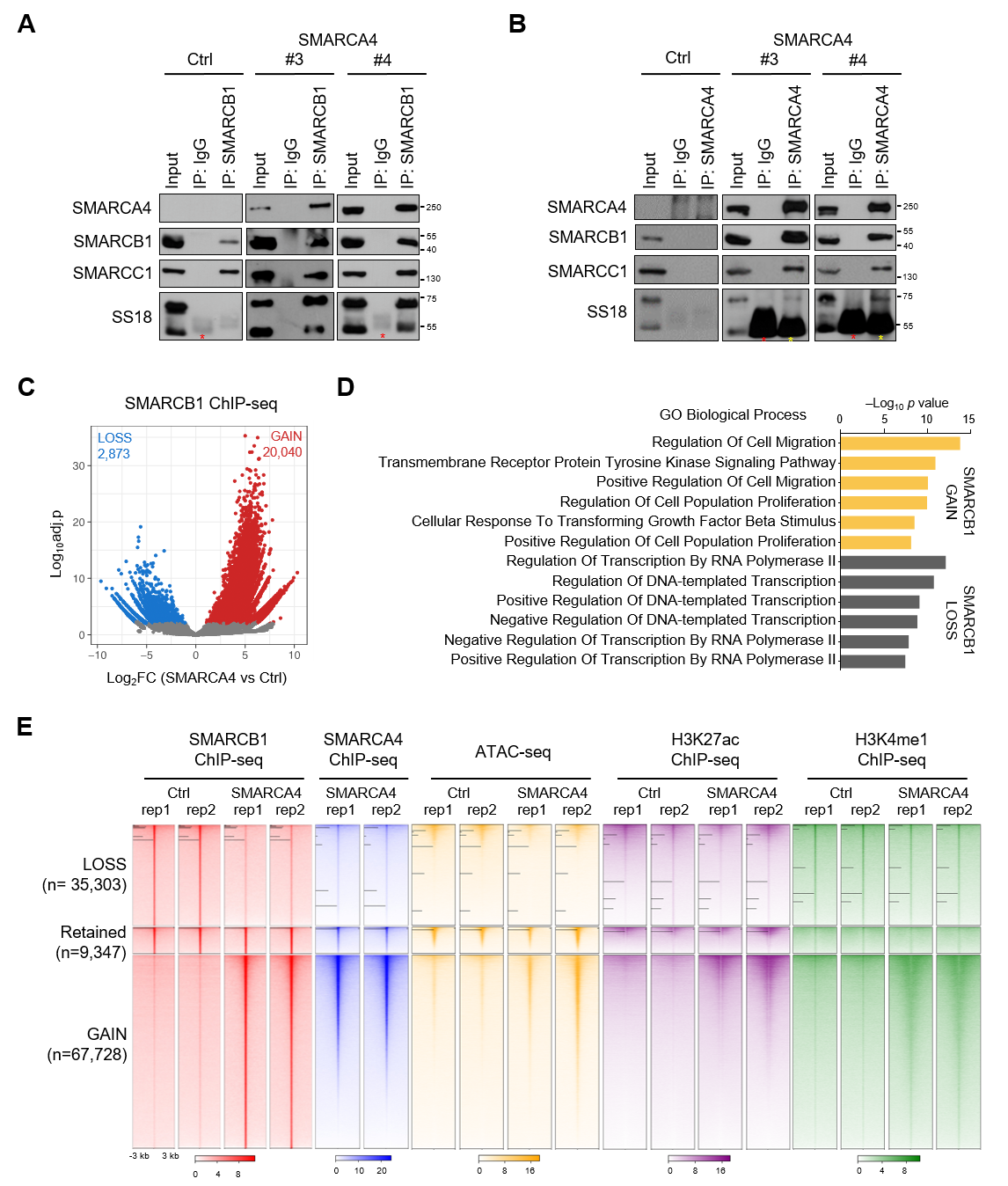
**


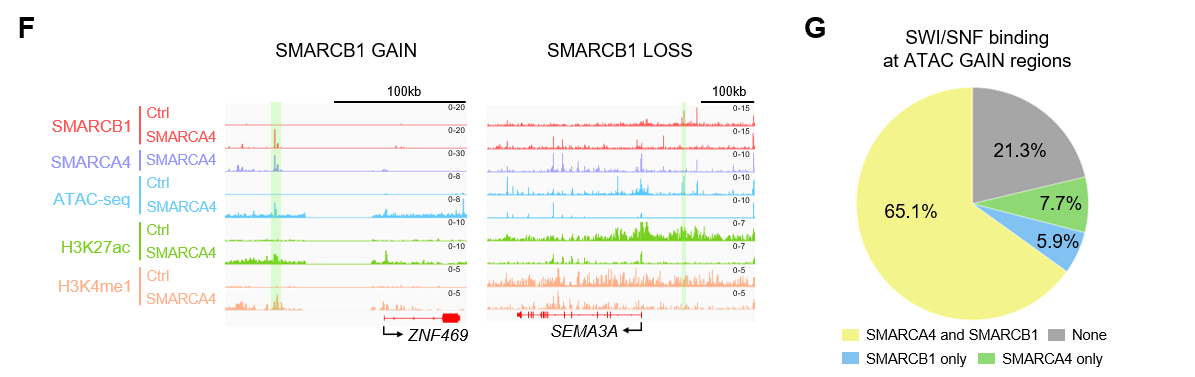


A, B. Western blot analysis of major SWI/SNF core subunits (SMARCA4, SMARCB1, SMARCC1) and SS18 in Ctrl cells and SMARACA4-expressing clone #4 following immunoprecipitation of SMARCB1 or SMARCA4. IgG bands are marked by red asterisks (*). The yellow asterisk (*) indicates SS18/IgG.

C. A volcano plot of differential SMARCB1 ChIP-seq peaks between Ctrl cells and SMARACA4 clone #4. FDR < 0.05, |log_2_FC| > 1. n = 2 biological replicates.

D. Top enriched gene ontology (GO) biological processes for the nearest genes of SMARCB1 GAIN and LOSS regions upon SMARCA4 overexpression.

E. Heatmaps of SMARCB1, SMARCA4, H3K27ac, H3K4me1 ChIP-seq signal and ATAC-seq signal in Ctrl cells and SMARACA4-expressing clone #4 at SMARCB1 LOSS, Retained, GAIN regions. n = 2 biological replicates.

F. IGV tracks of SMARCB1, SMARCA4, H3K27ac, and H3K4me1 enrichment (ChIP-seq) and chromatin accessibility (ATAC-seq) at the *ZNF469* (SMARCB1 GAIN) and *SEMA3A* loci (SMARCB1 LOSS) in Ctrl and SMARCA4-expressing clones.

G. Pie chart displaying the binding percentage of SMARCA4 and SMARCB1, SMARCB1 only, SMARCA4 only, or None (no SWI/SNF binding) at ATAC GAIN and LOSS regions.

**Supplementary Fig 2. SMARCA4 overexpression in SMARCA4-deficient LUAD cells leads to alterations in high-order chromatin structures**

**
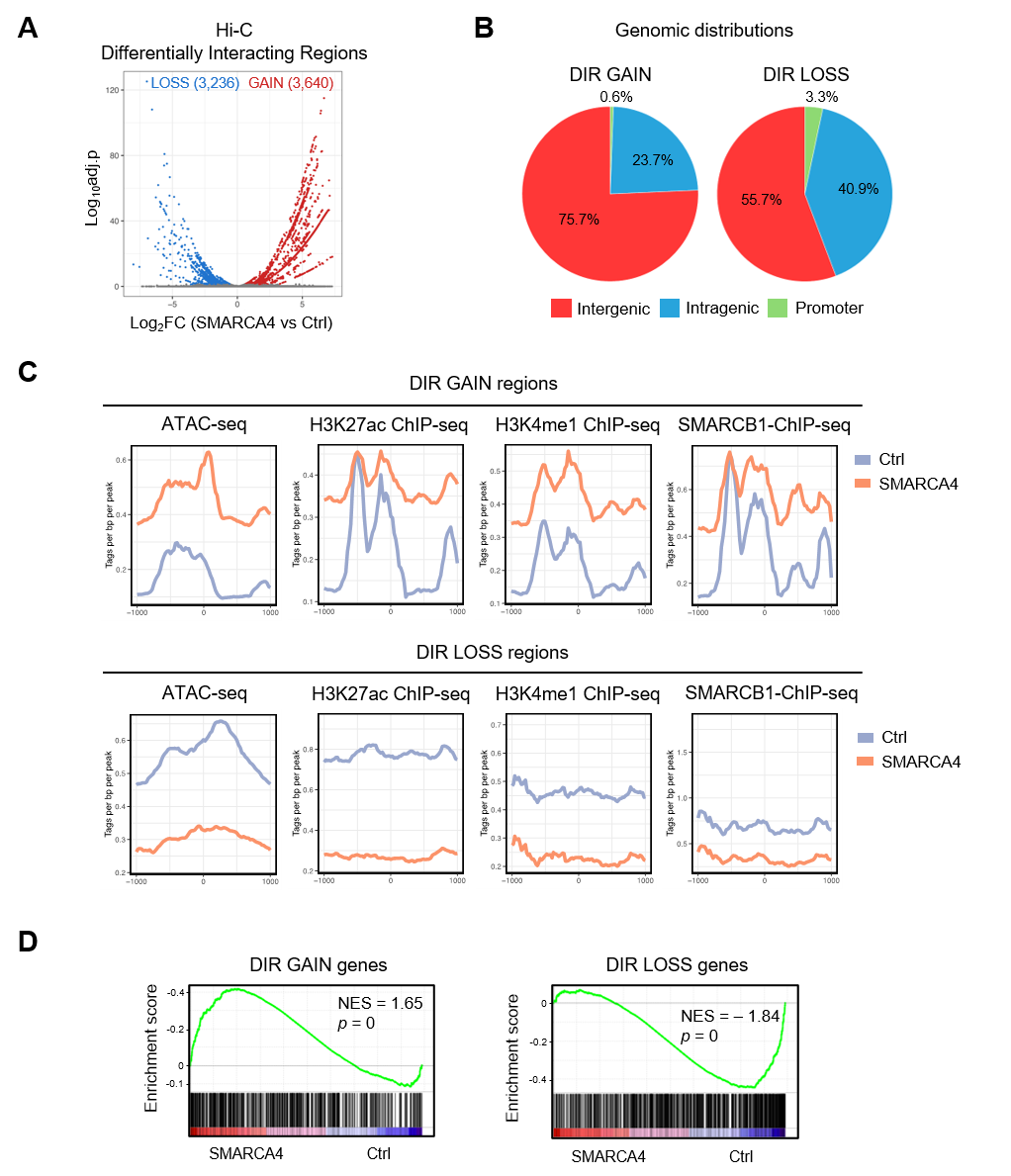
**

1. Volcano plot showing differentially interacting regions (DIRs) identified by Hi-C analysis between Ctrl cells and two independent clones expressing SMARCA4. n=2 biological replicates (Ctrl: two independent experiments; SMARCA4: clones #3 and #4). FDR < 0.05, Resolution = 1kb.
2. Pie charts displaying genomic distribution of DIR GAIN and LOSS regions. Intergenic; intergenic, Intragenic; exon, intron, transcription termination sites, Promoter; promoter.
3. Average plot of ATAC-seq signal and H3K27ac, H3K4me1, SMARCB1 ChIP-seq signal in Ctrl cell and SMARACA4-expressing clones at DIR GAIN (top) and LOSS (bottom) regions.
4. Gene set enrichment analysis (GSEA) of RNA-seq data from Ctrl cells and SMARACA4-expressing clones using a signature of DIR GAIN- or LOSS-associated genes. Normalized enrichment score (NES) and nominal *p*-value were provided according to GSEA.

**Supplementary Fig 3. SMARCA4 regulates GAIN enhancer-associated gene expression**

**
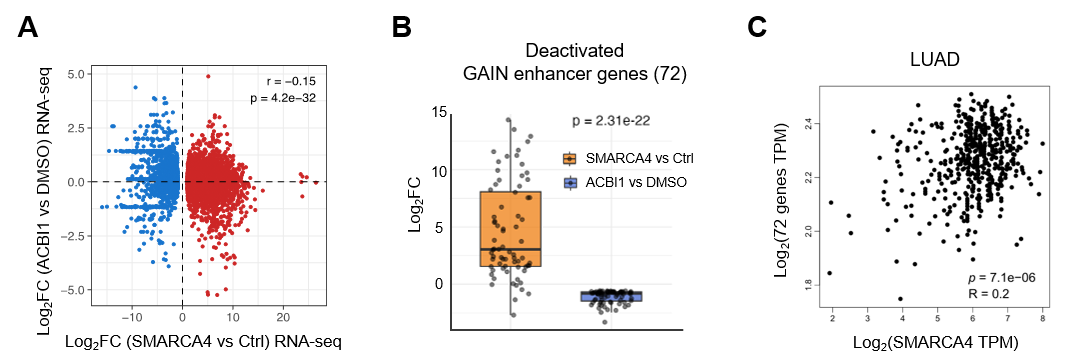
**

1. Scatter plots showing the correlation of gene expression change when SMARCA4 was overepxressed or ACBI1 was treated.
2. Box plot showing the expression changes for genes associated with deactivated GAIN enhancers (n = 72). Each dot represents an individual gene. *p*-values were calculated using the Wilcoxon rank-sum test.
3. Scatter plot showing positive correlation of SMARCA4 with deactivated GAIN Enhance genes in human TCGA-LUAD tumor dataset (n = 483) generated from GEPIA2. R, Pearson’s correlation coefficient.

**Supplementary Fig 4. FOSL1 depletion suppresses GAIN enhancer activity and target gene expression**

**
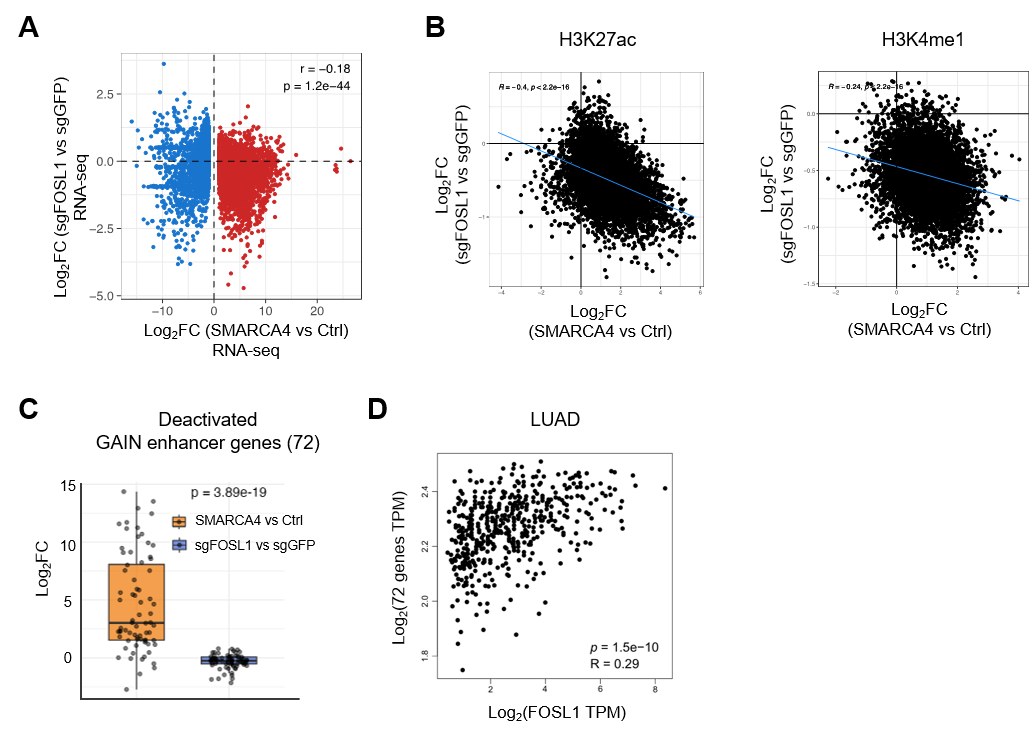
**

1. Scatter plot showing the correlation of gene expression change when SMARCA4 was overexpressed or FOSL1 was depleted by sgRNAs.
2. Scatter plot showing the correlation of H3K27ac (left) and H3K4me1 (right) ChIP-seq signal change when SMARCA4 was overexpressed or FOSL1 was depleted by sgRNAs.
3. Box plot showing the expression changes for genes associated with deactivated GAIN enhancers (n = 72). Each dot represents an individual gene. *p*-values were calculated using the Wilcoxon rank-sum test.
4. Scatter plot showing positive correlation of FOSL1 with deactivated GAIN enhancer genes in human TCGA-LUAD tumor dataset (n = 483) generated from GEPIA2. R, Pearson’s correlation coefficient.
