# Supplementary material for: SMARCA4 activation engages FOSL1 to drive enhancer reprogramming and tumorigenic phenotypes in SMARCA4-deficient LUAD cells
Source: Cell Death Discov. 2026 Apr 20;12:262. doi: 10.1038/s41420-026-03100-3 (PMC13223256; doi:10.1038/s41420-026-03100-3)
Supplement: Supplementary file 2 — Supplementary Tables [file 41420_2026_3100_MOESM2_ESM.docx]

**Supplementary Table 1. sgRNA sequences**

|  |  | sgRNA Sequences (5' → 3') |
| --- | --- | --- |
| sgGFP | F | CACCGGGGCGAGGAGCTGTTCACCG |
|  | R | AAACCGGTGAACAGCTCCTCGCCCC |
| sgFOSL1 | F | CACCGGGGCATGTTCCGAGACTTCG |
|  | R | AAACCGAAGTCTCGGAACATGCCCC |

**Supplementary Table 2. mRNA primer sequences**

| Target gene |  | mRNA primer sequences (5' → 3') |
| --- | --- | --- |
| FOSL1 | F | GCCCACTGTTTCTCTTGAGC |
|  | R | GGAGATAGGGTTGGGTGGAT |
| SOX9 | F | TTGAGCCTTAAAACGGTGCT |
|  | R | CTGGTGTTCTGAGAGGCACA |
| MET | F | CAGGCAGTGCAGCATGTAGT |
|  | R | GATGATTCCCTCGGTCAGAA |
| F3 | F | GGGCTGACTTCAATCCATGT |
|  | R | GAAGGTGCCCAGAATACCAA |
| FGFBP1 | F | CCTCAGCATAGTGCAGGACA |
|  | R | GCAGGAAACAGCCTCTGAAC |
| LAMB1 | F | AACGTGGTTGGAAGAACCTG |
|  | R | ACACTCCCTGGAAACAGTGG |
