# Supplementary material for: SMARCA4 activation engages FOSL1 to drive enhancer reprogramming and tumorigenic phenotypes in SMARCA4-deficient LUAD cells
Source: Cell Death Discov. 2026 Apr 20;12:262. doi: 10.1038/s41420-026-03100-3 (PMC13223256; doi:10.1038/s41420-026-03100-3)

# Original Western Blots

# Figure 1

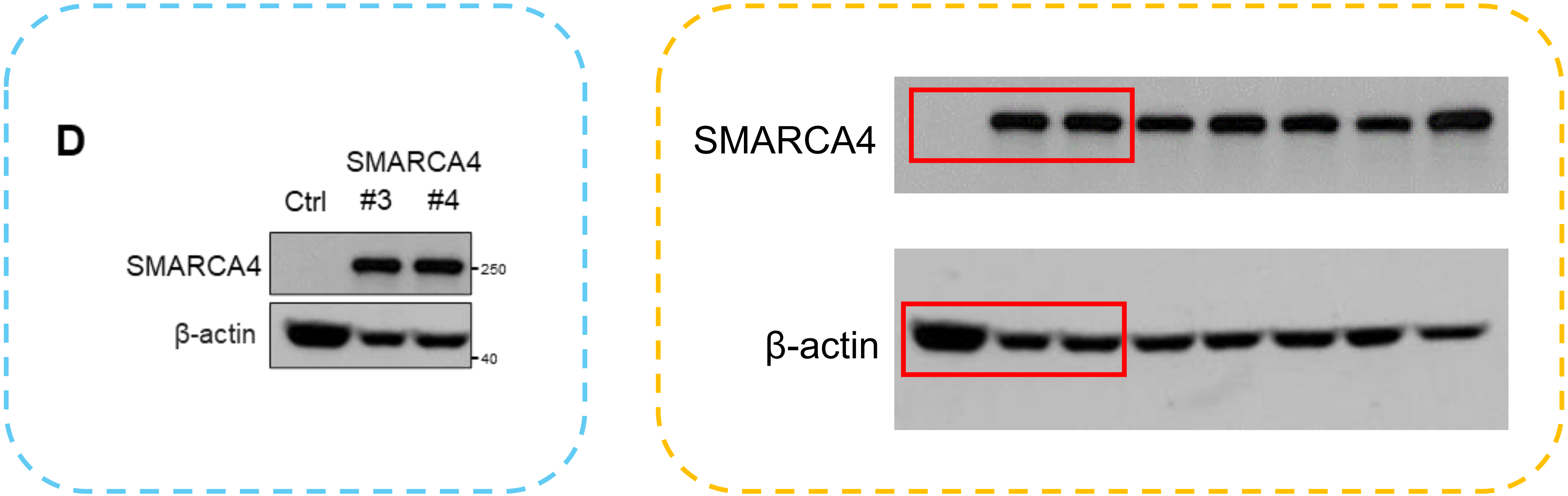

# Figure 4

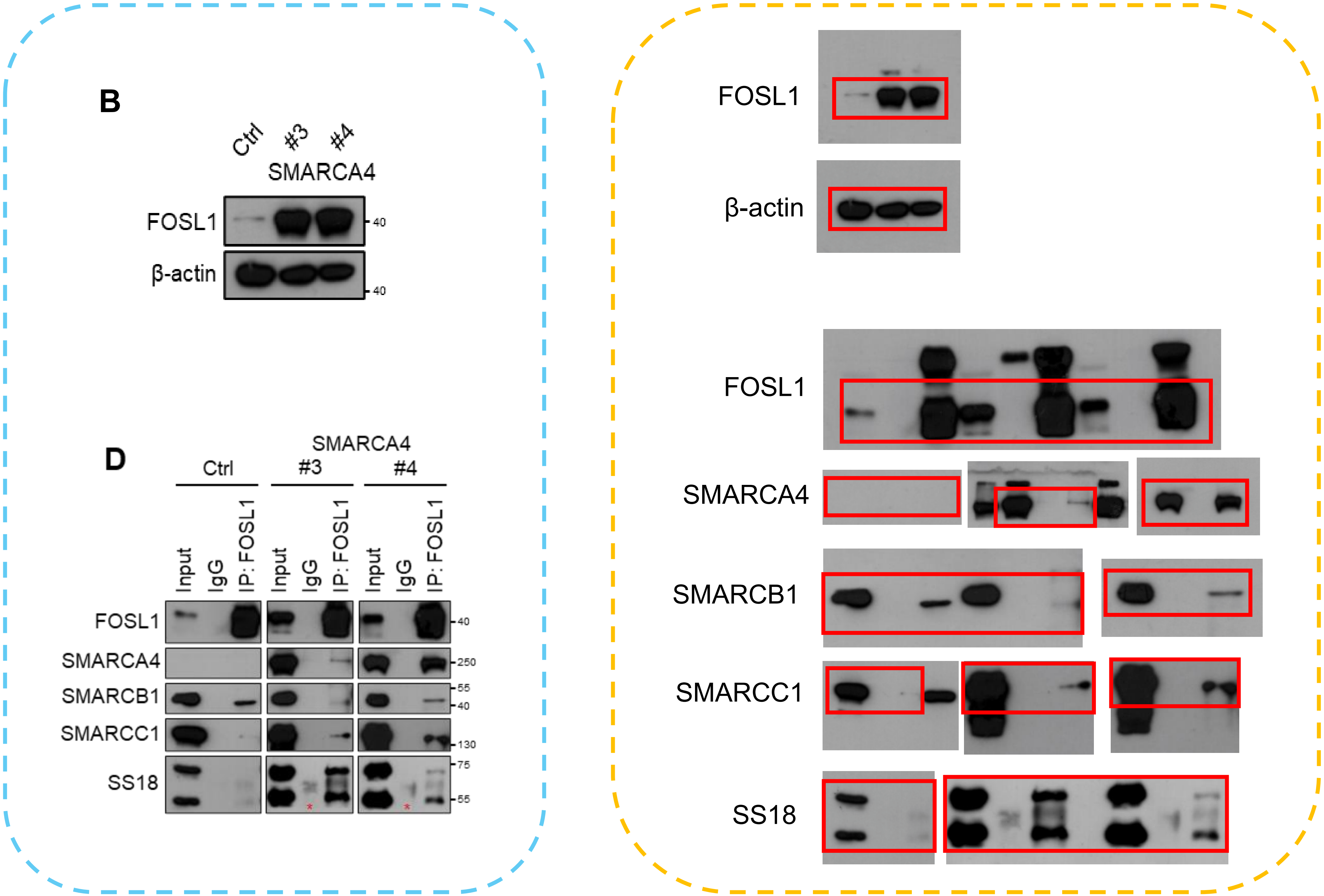
B
Ctrl
#3
#4
SMARCA4
FOSL1
β-actin
40
40
D
SMARCA4
Ctrl
#3
#4
Input
IgG
IP: FOSL1
FOSL1
SMARCA4
SMARCB1
SMARCC1
SS18
250
55
130
75
FOSL1
β-actin
FOSL1
SMARCA4
SMARCB1
SMARCC1
SS18

# Figure 5

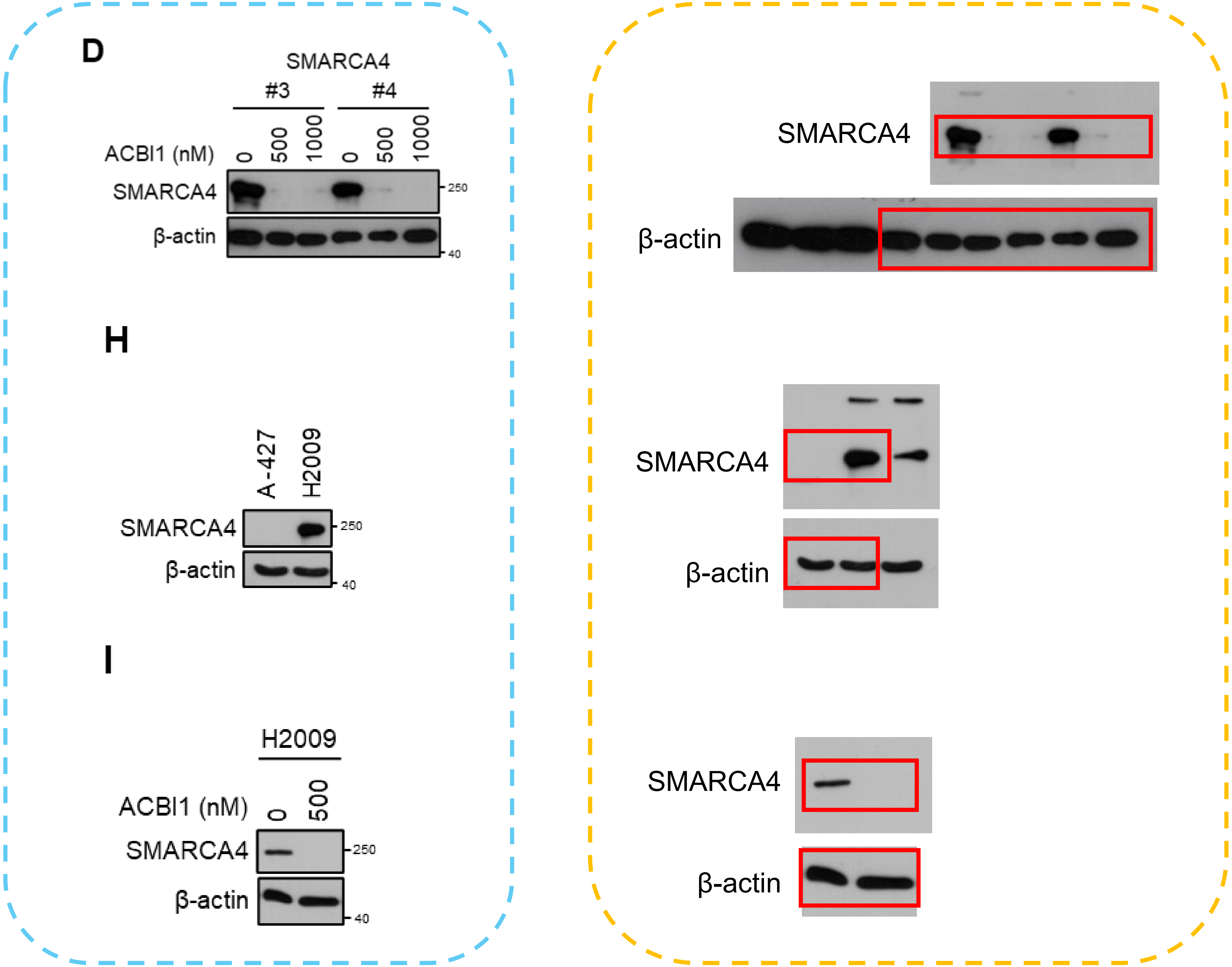
D
SMARCA4
#3
#4
ACBI1 (nM)
0
500
1000
0
500
1000
SMARCA4
β-actin
250
40
H
A-427
H2009
SMARCA4
β-actin
250
40
I
H2009
ACBI1 (nM)
0
500
SMARCA4
β-actin
250
40
SMARCA4
β-actin
SMARCA4
β-actin
SMARCA4
β-actin

# Figure 7

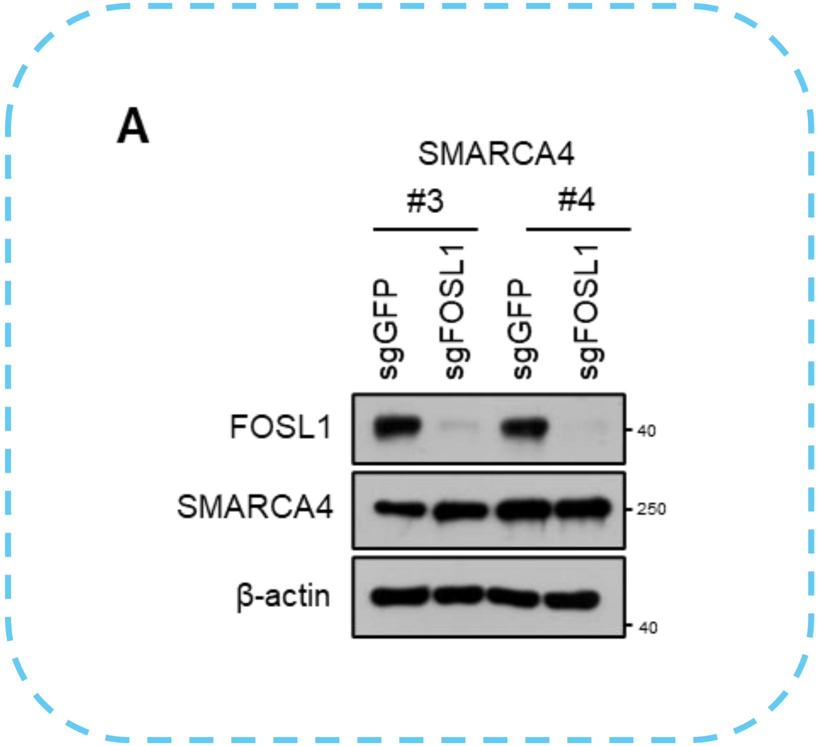
A
SMARCA4
#3
#4
sgGFP
sgFOSL1
sgGFP
sgFOSL1
FOSL1
SMARCA4
β-actin
40
250
40

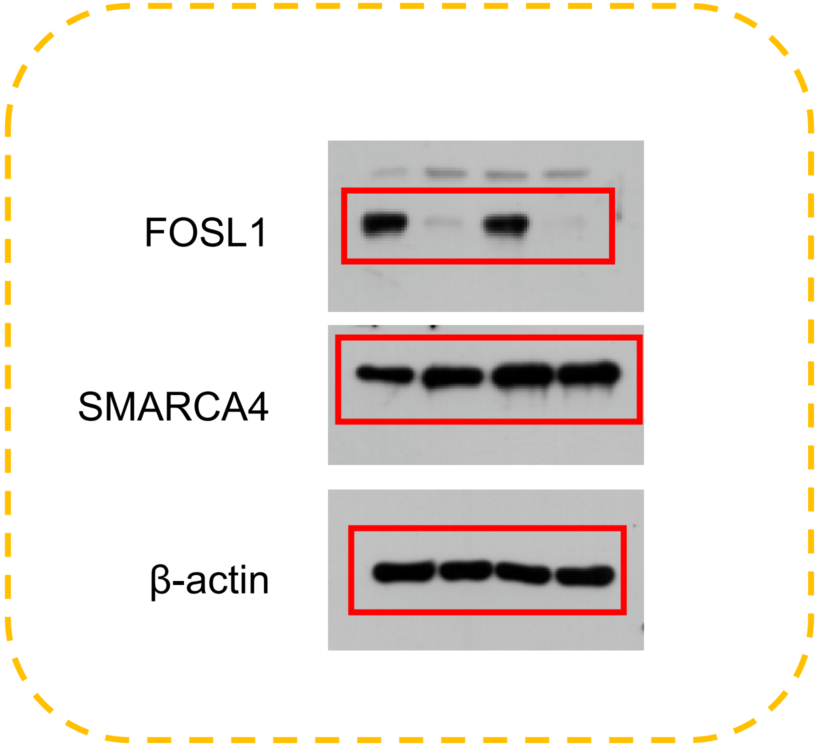
FOSL1
SMARCA4
β-actin

# Figure 8

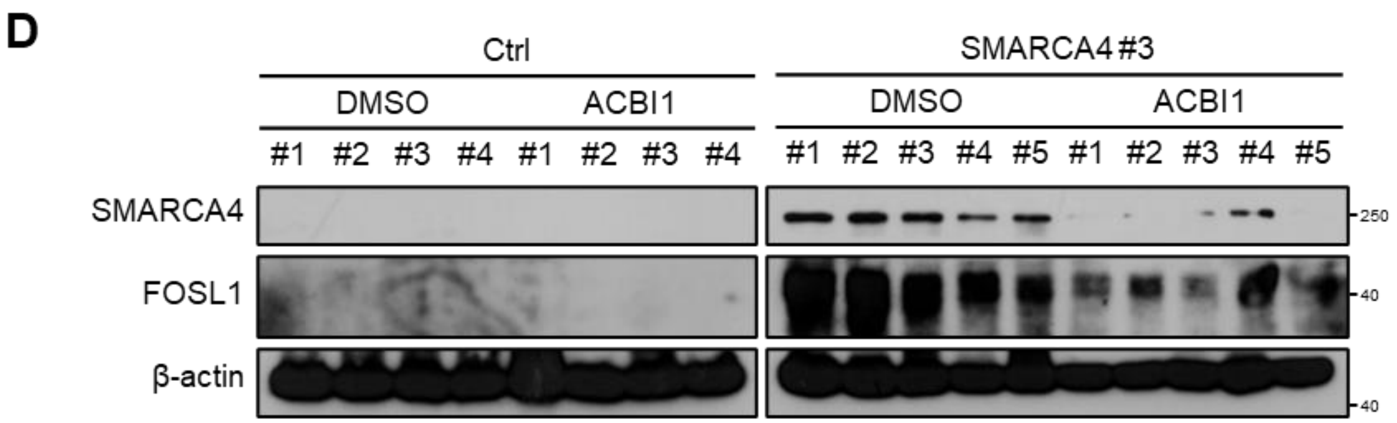
D
Ctrl
SMARCA4 #3
DMSO
ACBI1
DMSO
ACBI1
#1 #2 #3 #4 #1 #2 #3 #4
#1 #2 #3 #4 #5 #1 #2 #3 #4 #5
SMARCA4
FOSL1
β-actin
250
40
40

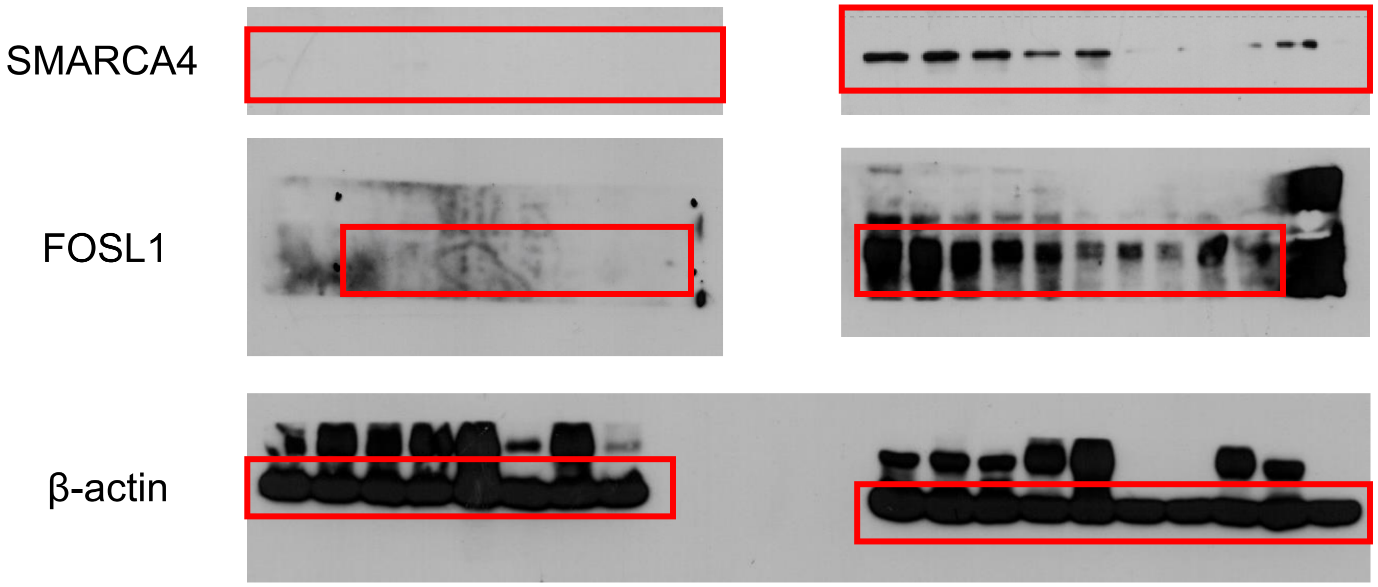
SMARCA4
FOSL1
β-actin

# Supplementary Figure 1

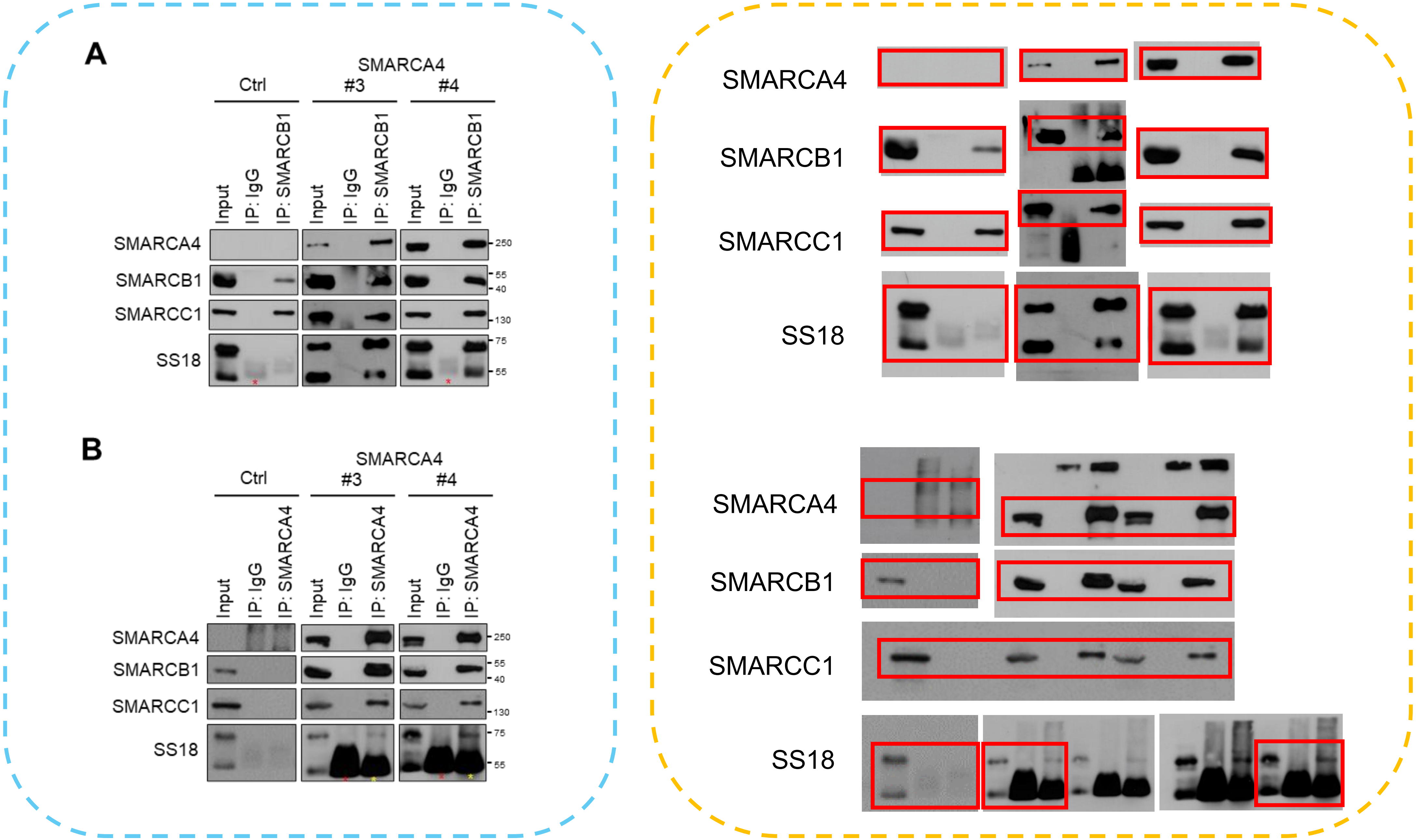

Supplement: Supplementary file 3 — Original western blot [file 41420_2026_3100_MOESM3_ESM.pdf]
